# Directing 2D-Coordination Networks: Combined Effects of a Conformationally Flexible 3,2′:6′,3″-Terpyridine and Chain Length Variation in 4′-(4-*n*-Alkyloxyphenyl) Substituents

**DOI:** 10.3390/molecules25071663

**Published:** 2020-04-04

**Authors:** Dalila Rocco, Alessandro Prescimone, Edwin C. Constable, Catherine E. Housecroft

**Affiliations:** Department of Chemistry, University of Basel, BPR 1096, Mattenstrasse 24a, CH-4058 Basel, Switzerland; dalila.rocco@unibas.ch (D.R.); alessandro.prescimone@unibas.ch (A.P.); edwin.constable@unibas.ch (E.C.C.)

**Keywords:** 3,2′:6′,3″-terpyridine, 2D-coordination network, cobalt(II) thiocyanate

## Abstract

The synthesis and characterization of 4′-(4-*n*-propoxyphenyl)-3,2′:6′,3″-terpyridine is described. Five 2D-coordination networks have been isolated by crystal growth at room temperature from reactions of Co(NCS)_2_ with 4′-(4-*n*-alkyloxyphenyl)-3,2′:6′,3″-terpyridines in which the *n*-alkyl group is ethyl, *n*-propyl, *n*-butyl, *n*-pentyl and *n*-hexyl in ligands **2**–**6**, respectively. The single-crystal structures of [{Co(**2**)_2_(NCS)_2_}**^.^**0.6CHCl_3_]*_n_*, [{Co(**3**)_2_(NCS)_2_}**^.^**4CHCl_3_**^.^**0.25H_2_O]*_n_*, [{Co(**4**)_2_(NCS)_2_}**^.^**4CHCl_3_]*_n_*, [Co_2_(**5**)_4_(NCS)_4_]*_n_* and [Co(**6**)_2_(NCS)_2_]*_n_* have been determined, and powder X-ray diffraction has demonstrated that the single-crystal structures are representative of the bulk materials. Each compound possesses a (4,4) net with Co centres as 4-connecting nodes. For the assemblies containing **2**, **3** and **4**, the (4,4) net comprises two geometrically different rhombuses, and the nets pack in an ABAB... arrangement with cone-like arrangements of *n*-alkyloxyphenyl groups being accommodated in a similar unit in an adjacent net. An increase in the *n*-alkyloxy chain length has two consequences: there is a change in the conformation of the 3,2′:6′,3″-tpy metal-binding domain, and the (4,4) net comprises identical rhombuses. Similarities and differences between the assemblies with ligands **2**–**6** and the previously reported [{Co(**1**)_2_(NCS)_2_}**^.^**3MeOH]*_n_* and [{Co(**1**)_2_(NCS)_2_}**^.^**2.2CHCl_3_]*_n_* in which **1** is 4′-(4-methoxyphenyl)-3,2′:6′,3″-terpyridine are discussed. The results demonstrate the effects of combining a variable chain length in the 4′-(4-*n*-alkyloxyphenyl) substituents of 3,2′:6′,3″-tpy and a conformationally flexible 3,2′:6′,3″-tpy metal-binding domain.

## 1. Introduction

**Scheme 1:**
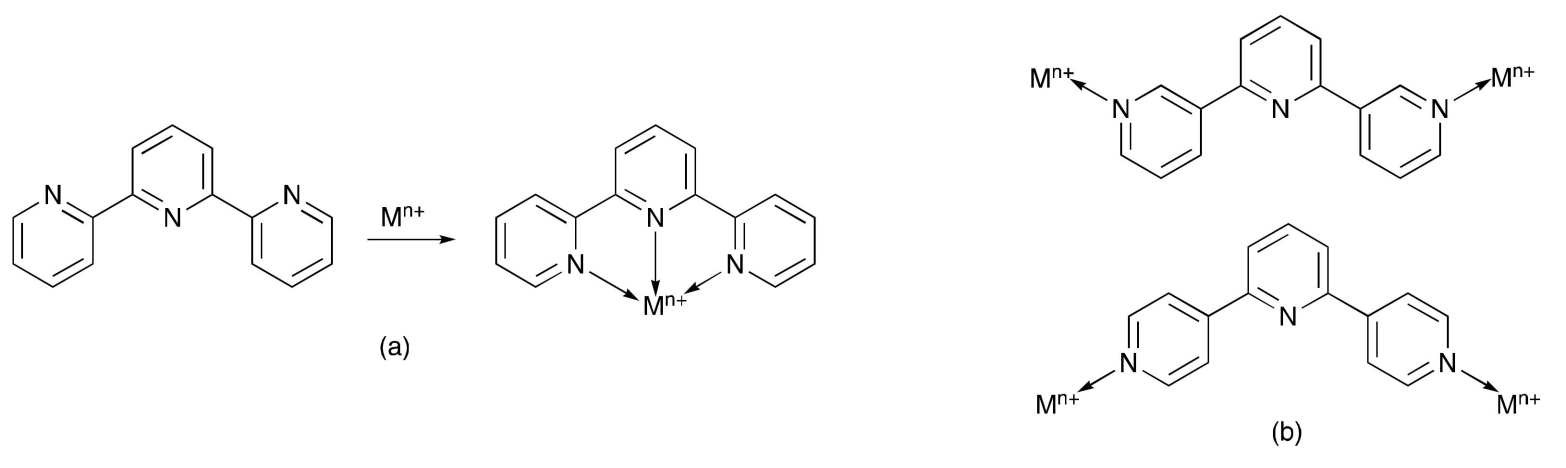
(**a**) Conformational change of 2,2′:6′,2″-tpy upon coordination to a metal ion. (**b**) Both 3,2′:6′,3″-tpy and 4,2′:6′,4″-tpy coordinate only through the outer pyridine rings.

Terpyridine (tpy) possesses 48 possible isomers, of which 2,2′:6′,2″-tpy is the most commonly encountered [1,2,3,4,5]. Coordination to a metal usually involves a conformational change from the limiting *trans,trans*-form found for the free ligand to the limiting *cis*,*cis*-conformer as two chelate rings are formed (Scheme 1a). Nonetheless, there is a significant number of metal coordination compounds of 2,2′:6′,2″-tpy that do not involve a chelating tridentate mode [6]. The isomers 3,2′:6′,3″-terpyridine (3,2′:6′,3″-tpy) and 4,2′:6′,4″-terpyridine (4,2′:6′,4″-tpy), along with their 4′-functionalized derivatives, are synthetically accessible by using either the Kröhnke strategy [7] or Wang and Hanan’s one-pot method [8]. In contrast to 2,2′:6′,2″-tpy, which is usually monotopic, 3,2′:6′,3″-tpy and 4,2′:6′,4″-tpy typically coordinate to two metal centres and are ditopic ligands (Scheme 1b). Searches of the Cambridge Structural Database (CSD, v. 5.4.1 [9]) do not reveal any examples of metal complexes in which the central pyridine ring of 3,2′:6′,3″-tpy and 4,2′:6′,4″-tpy binds a metal ion. The 3,2′:6′,3″-tpy and 4,2′:6′,4″-tpy metal-binding domains differ in their degrees of conformational flexibility [10]. We recently demonstrated that in a series of 1D-coordination polymers [Cu_2_(μ-OAc)_4_(L)]*_n_* in which L is a 4′-(4-*n*-alkyloxyphenyl)-substituted 3,2′:6′,3″-tpy ligand, conformational changes of the 3,2′:6′,3″-tpy domain associated with an increase in the length of the *n*-alkyloxy substituent results in significant changes in the conformation and packing of the coordination polymer chains [11].

The use of 4,2′:6′,4″-tpy ligands, often functionalized in the 4′-position, as building blocks in coordination polymers and networks has gained in popularity over the last decade [12,13]. In contrast, networks incorporating 3,2′:6′,3″-tpy metal-binding domains are significantly fewer in number. A coordination network is defined by a combination of nodes and linkers. Three scenarios are possible: metal nodes and organic linkers, ligand nodes and metal linkers, or a combination of metal and organic ligand nodes. Ditopic ligands such as 4,2′:6′,4″-tpy and 3,2′:6′,3″-tpy are restricted to roles as linkers, unless functionalized with one or more additional donor groups. However, in contrast to predetermined structural assemblies made possible by the choice of rigid-rod linkers such as 4,4′-bipyridine [14,15], the conformational flexibility of 3,2′:6′,3″-tpy makes predictive network assembly more challenging. This makes systematic investigations all the more important. For 4′-(4-*n*-alkyloxyphenyl)-substituted 4,2′:6′,4″-tpy ligands, systematically lengthening the alkyloxy substituents results in a switch from 1D-coordination polymers [Zn_2_(μ-OAc)_4_(4′-(4-alkyloxyphenyl)-4,2′:6′,4″-tpy)]*_n_* (alkyl = methyl, ethyl, *^n^*propyl) to discrete [Zn_2_(μ-OAc)_4_(4′-(4-alkyloxyphenyl)-4,2′:6′,4″-tpy)_2_] complexes (alkyl = *n*-octyl, *n*-nonyl, *n*-decyl) with a region of competition between these extremes [16]. Another example of the redirection of an assembly by going from long to short alkyloxy substituents is a switch from 2D→2D parallel interpenetration of (4,4) sheets to single (4,4) nets as the stabilizing influence of long *n-*octyl chains is removed [17,18].

**Scheme 2:**
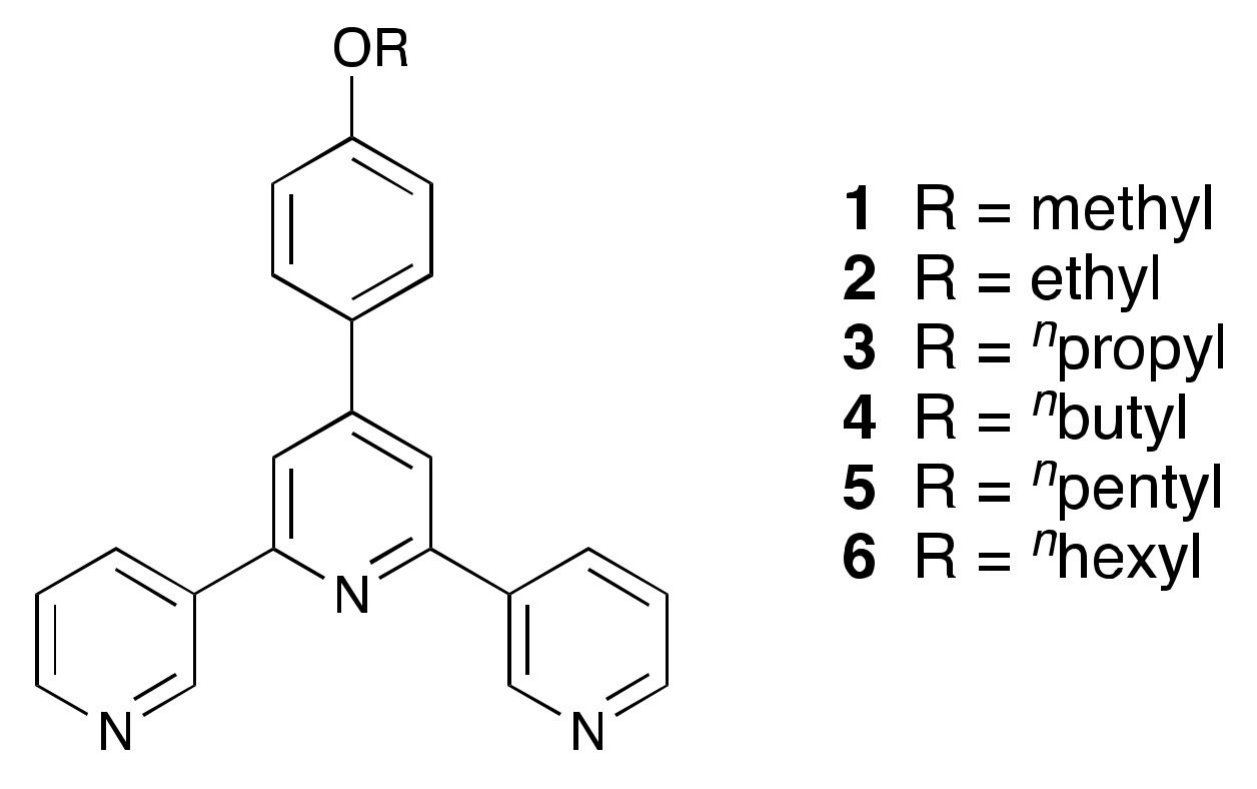
Structures of ligands **1**–**6**.

In a continuation of our investigations of the coordination of 4′-(4-alkyloxyphenyl)-3,2′:6′,3″-tpy ligands [11,19], we now explore the effects of increasing the length of the alkyloxy substituent from methoxy to *^n^*hexyloxy (ligands **1**–**6**, Scheme 2). We have previously shown that crystal growth by layering a methanol solution of Co(NCS)_2_ over a chloroform solution of **1** yields either [Co(**1**)(NCS)_2_(MeOH)_2_]*_n_* (1D-chain), [{Co(**1**)_2_(NCS)_2_}**^.^**3MeOH]*_n_* ((4,4)-net) or [{Co(**2**)_2_(NCS)_2_}**^.^**2.2CHCl_3_]*_n_* ((4,4)-net), depending on the crystallization conditions. However, powder X-ray diffraction (PXRD) revealed that in experiments where crystals selected for single crystal X-ray diffraction proved to be the (4,4)-nets, the 1D-polymer [Co(**1**)(NCS)_2_(MeOH)_2_]*_n_* was always the dominant product [19]. 

## 2. Results and discussion

### 2.1. Synthesis and Characterization of Compound **3**

**Scheme 3:**
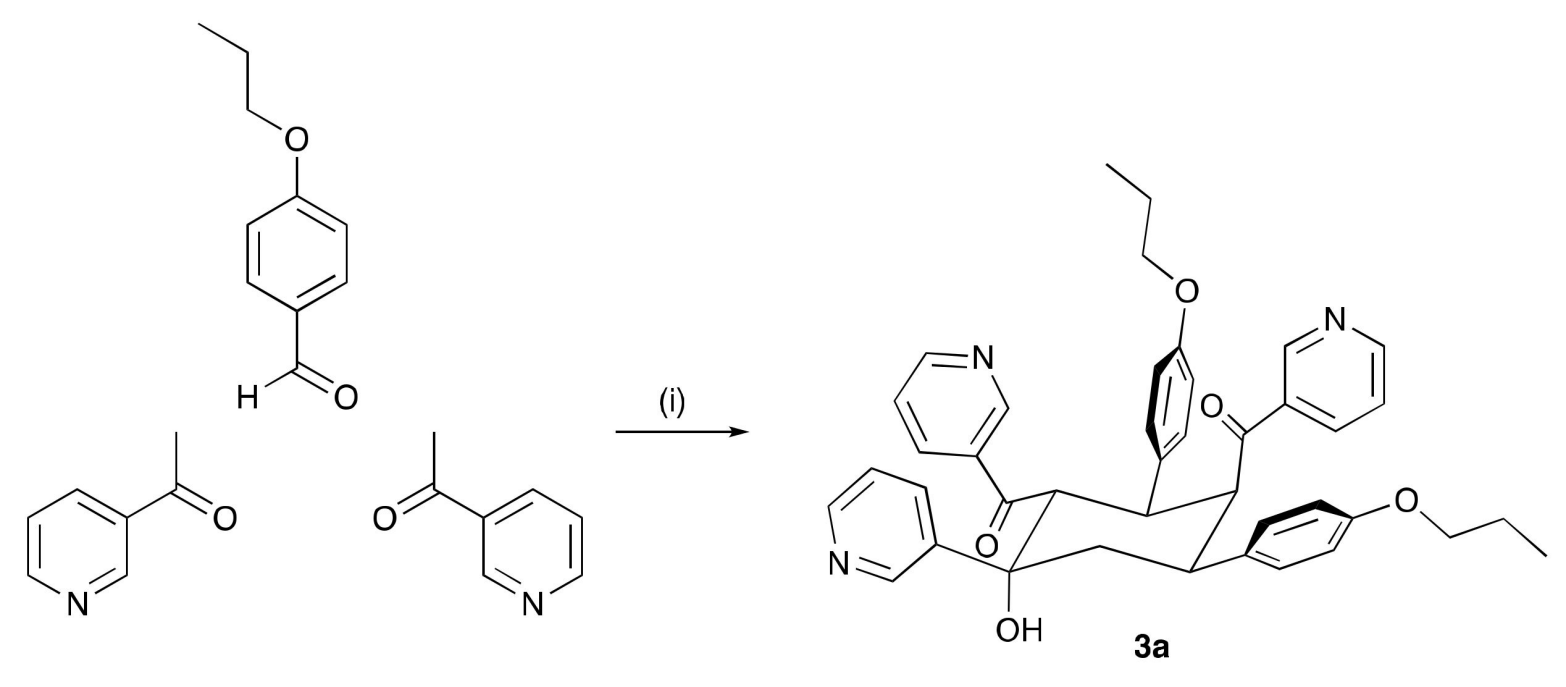
The previously reported attempt to prepare **3** led to the cyclic product **3a** [20].

**Scheme 4:**
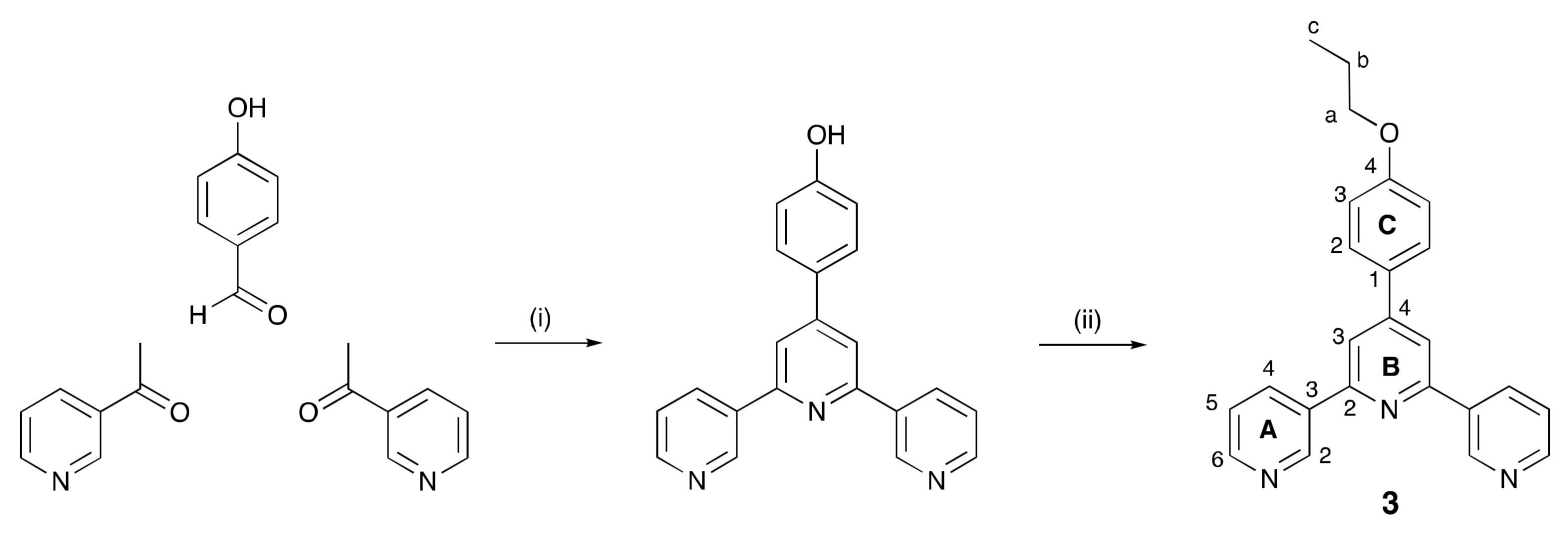
Synthetic route to compound **3**. Conditions: (i) KOH, EtOH; NH_3_ (aqueous) pH 8. (ii) DMF, K_2_CO_3_; 1-bromopropane, 70 °C, overnight.

**Figure 1:**
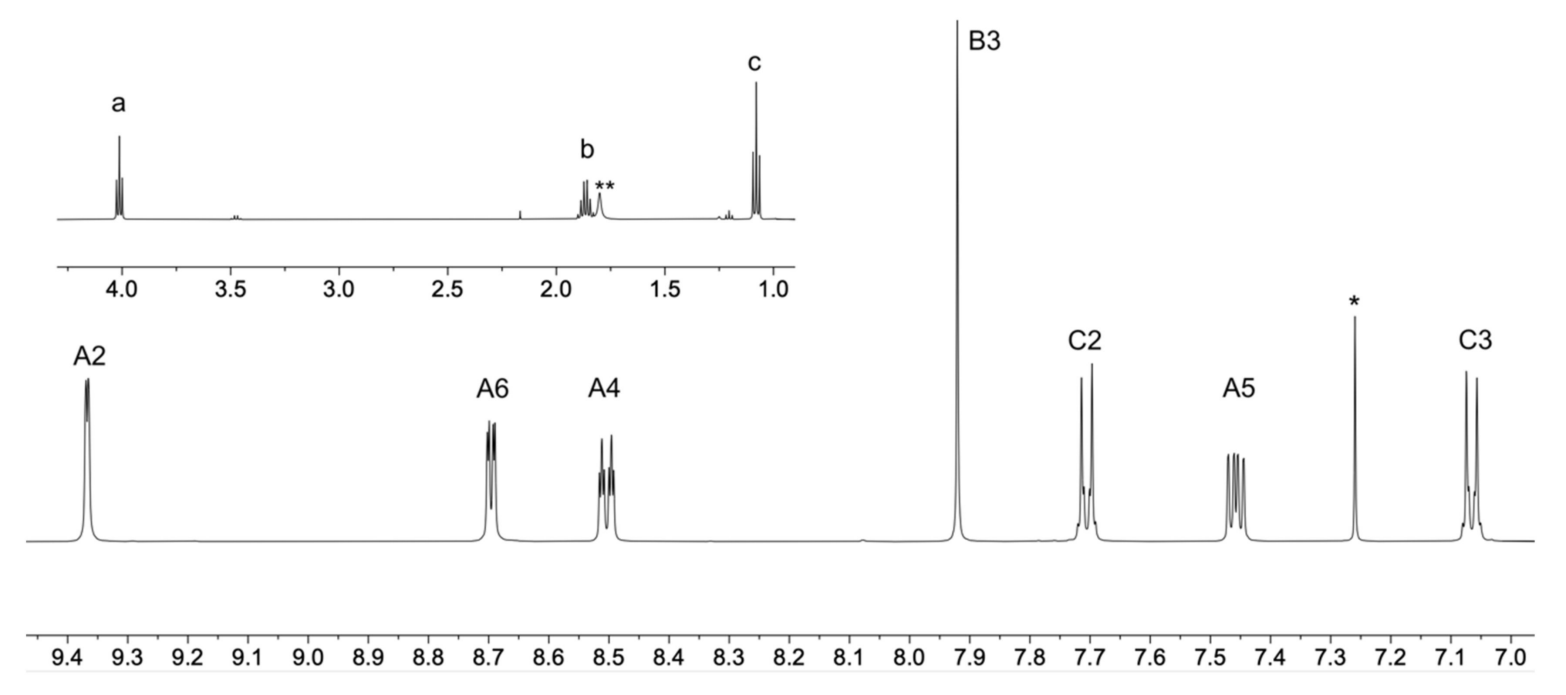
The aromatic region of the ^1^H NMR spectrum (500 MHz, CDCl_3_, 298 K) of compound **3** with the alkyl region shown in the inset. Scale: *δ*/ppm. * = residual CHCl_3_. See Scheme 4 for atom labels.

A well-established route to 4′-aryl functionalized 2,2′:6′,2″-terpyridines is the one-pot strategy of Wang and Hanan [8], and this methodology has also been used to prepare the 3,2′:6′,3″-terpyridines **1** [19], **2** [20] and **4**–**6** [11,20,21]. However, in the case of compound **3**, this strategy failed [20] and led instead to the cyclic product **3a** (Scheme 3). We therefore adopted the approach shown in Scheme 4 to synthesize compound **3**. We first prepared 4′-(4-hydroxyphenyl)-3,2′:6′,3″-terpyridine, choosing the Wang and Hanan approach [8] rather than the solventless method reported by Cave and Raston [22]. The reaction of 4′-(4-hydroxyphenyl)-3,2′:6′,3″-terpyridine with 1-bromopropane (Scheme 4) resulted in the formation of **3** in a 37.8% yield after purification. The base peak in the electrospray (ESI) mass spectrum of **3** at *m/z* 368.15 arose from [M+H]^+^, and both this and the high-resolution ESI mass spectrum are displayed in Figure S1. The ^1^H NMR spectrum of compound **3** (Figure 1) is consistent with the structure shown in Scheme 2. Figures S2–S6 in the supporting information display the ^1^H, ^13^C{^1^H} NMR and solid-state IR spectra of **3**, and NMR assignments (see Section 3.2) were made using 2D methods. 

**Figure 2:**
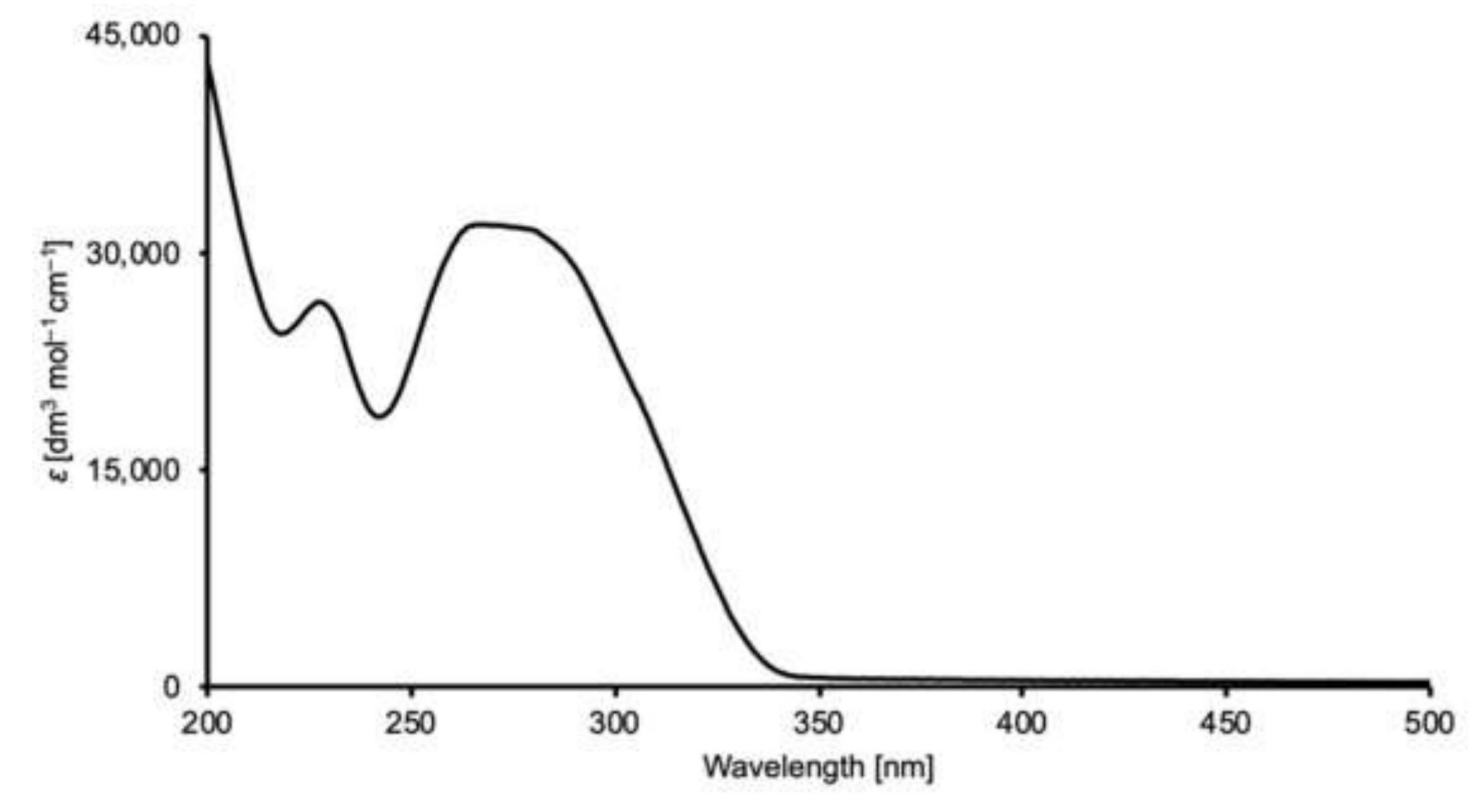
The solution absorption spectrum of compound **3** (MeCN, 2 × 10^−3^ mol dm^−3^).

The solution absorption spectrum of **3** (Figure 2) is reminiscent of the spectra of other members of the homologous series of ligands shown in Scheme 2 [11,19,20], and the absorptions are assigned to a combination of spin-allowed π*←*n* and π*←π transitions. 

### 2.2. Reactions of Compounds **2–6** with Co(NCS)_2_ and Bulk Material Characterization

**Figure 3:**
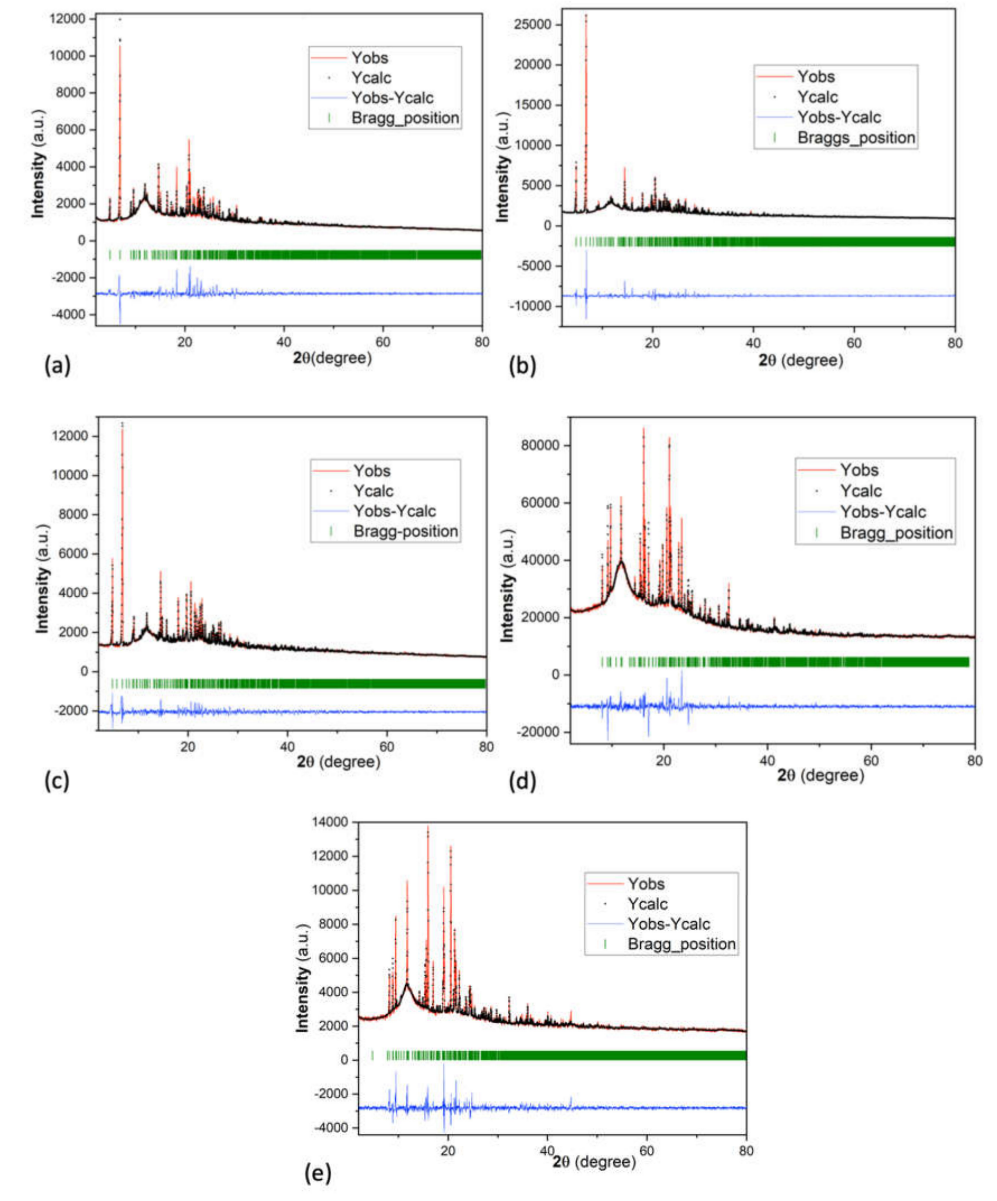
Laboratory powder X-Ray diffraction (CuKα1 radiation, 298 K) pattern (red trace) of each bulk crystalline material from which single crystals of (**a**) [{Co(**2**)_2_(NCS)_2_}**^.^**0.6CHCl_3_]*_n_*, (**b**) [{Co(**3**)_2_(NCS)_2_}**^.^**4CHCl_3_**^.^**0.25H_2_O]*_n_*, (**c**) [{Co(**4**)_2_(NCS)_2_}**^.^**4CHCl_3_]*_n_*, (**d**) [Co_2_(**5**)_4_(NCS)_4_]*_n_* and (**e**) [Co(**6**)_2_(NCS)_2_]*_n_* were selected. Each experimental powder pattern was fitted to the predicted pattern from the single crystal determination. The black dots correspond to the best fit from the profile matching refinement. Lower vertical green marks denote the Bragg peak positions. The bottom (blue) line in each plot represents the difference between the experimental and calculated points.

Methanol solutions of cobalt(II) thiocyanate were layered over chloroform solutions of ligands **2**–**6** under the same room temperature conditions. For each ligand, the reactions were carried out using metal:ligand molar ratios of 1:1, 2:1 and 1:2, and the conditions detailed in Section 3.2, Section 3.3, Section 3.4, Section 3.5 and Section 3.6 correspond to the setups in which crystals grew. Irrespective of the initial metal:ligand ratios, the crystalline products were coordination networks in which the Co:ligand ratio was 1:2. After selection of single crystals for X-ray diffraction, the bulk materials were analyzed by PXRD and solid-state IR spectroscopy, and where multiple experiments were carried out (see Section 3.2, Section 3.3 and Section 3.4), cell checks were carried out on single crystals from different batches. Powder patterns were fitted to those predicted from the single-crystal structures (Figure 3), and the data confirmed that crystals selected for single-crystal diffraction analysis were representative of the bulk samples for each of the coordination networks discussed in the next section. The fingerprint regions of the IR spectra of single crystals or microcrystalline samples (Figures S7–S11 in the supporting information) are all similar, consistent with analogous coordination behaviors of ligands **2**–**5** in the coordination assemblies. Each spectrum exhibits bands in the region between 2820 and 3100 cm^−1^ characteristic of the aromatic C–H stretches of the ligands. A strong absorption band in each IR spectrum at 2068 cm^−1^ (compounds with **2** and **3**), 2062 cm^−1^ (with **4**), 2074 cm^−1^ (with **5**) and 2072 cm^−1^ (with **6**) arises from the coordinated thiocyanate ligands.

### 2.3. Crystal Structures

Single crystal X-ray diffraction confirmed that the pink crystals grown by layering comprised [{Co(**2**)_2_(NCS)_2_}**^.^**0.6CHCl_3_]*_n_*, [{Co(**3**)_2_(NCS)_2_}**^.^**4CHCl_3_**^.^**0.25H_2_O]*_n_*, [{Co(**4**)_2_(NCS)_2_}**^.^**4CHCl_3_]*_n_*, [Co_2_(**5**)_4_(NCS)_4_]*_n_* and [Co(**6**)_2_(NCS)_2_]*_n_*. The compounds incorporating ligands **2**, **3** and **4** (ethoxy, *n*-propoxy and *n-*butoxy, respectively) crystallize in the tetragonal space groups *P*4*/ncc* or *P–*42_1_*c* (see Section 3.8, Section 3.9 and Section 3.10), while those containing ligands **5** and **6** (*n*-pentyloxy and *n*-hexyloxy) crystallize in the monoclinic space groups *P*2_1_*/n* and *P*2_1_*/c*, respectively. ORTEP diagrams of the asymmetric units with symmetry-generated atoms are displayed in Figures S12–S16. We discuss the structures together and focus on differences that correlate to the lengthening of the *n*-alkyloxy chain. In all the compounds, each cobalt atom is in an octahedral environment with a *trans*-arrangement of thiocyanato ligands and is bonded to one pyridine ring of four different 3,2′:6′,3″-tpy units. In each of [{Co(**3**)_2_(NCS)_2_}**^.^**4CHCl_3_**^.^**0.25H_2_O]*_n_*, [{Co(**4**)_2_(NCS)_2_}**^.^**4CHCl_3_]*_n_* and [Co(**6**)_2_(NCS)_2_]*_n_*, there are two independent ligands. Bond parameters for the coordination spheres of the cobalt atoms are compared in Table 1 and are unexceptional. In keeping with expectations, the central pyridine ring of each ligand is non-coordinated. 

**Scheme 5:**
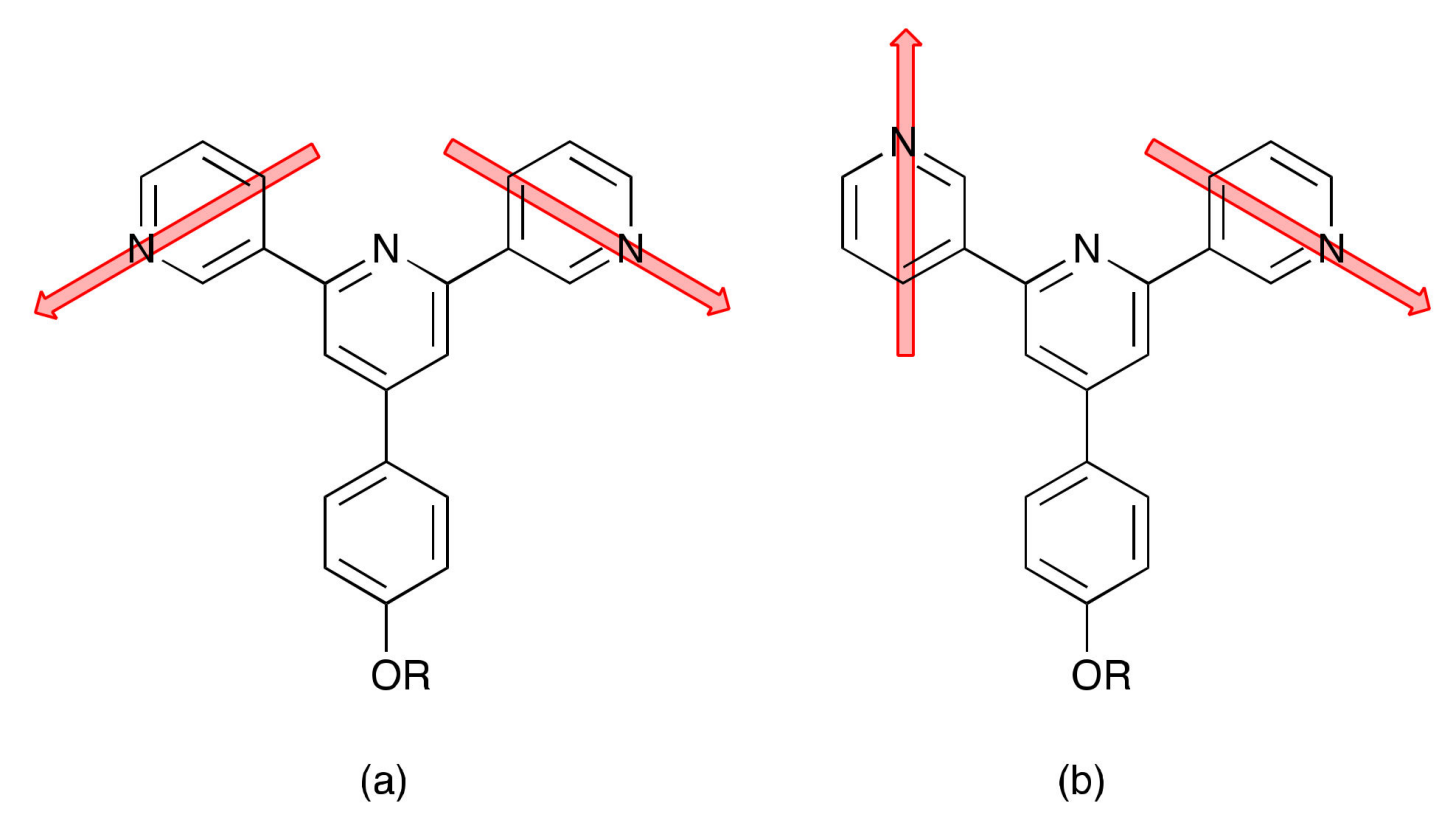
The ligand conformations found in the structures of (**a**) [{Co(**2**)_2_(NCS)_2_}**^.^**0.6CHCl_3_]*_n_*, [{Co(**3**)_2_(NCS)_2_}**^.^**4CHCl_3_**^.^**0.25H_2_O]*_n_* and [{Co(**4**)_2_(NCS)_2_}**^.^**4CHCl_3_]*_n_*, and (**b**) [Co_2_(**5**)_4_(NCS)_4_]*_n_* and [Co(**6**)_2_(NCS)_2_]*_n_*.

In each compound, the cobalt atom acts as a four-connecting node, and adjacent metal centers are bridged by 3,2′:6′,3″-tpy units to produce (4,4) nets. This is reminiscent of the coordination networks assembled from Co(NCS)_2_ and 4,2′:6′,4″-tpy ligands functionalized with 4′-*tert*-butyl, 4′-(4-methoxyphenyl), 4′-(4-ethoxyphenyl) and 4′-(4-*n*-propoxyphenyl) substituents [23]. However, the formation of a (4,4) net in [Co(**6**)_2_(NCS)_2_]*_n_* with the *n*-hexyloxy-tailed ligand **6** contrasts with the assembly of a 3D-framework when 4′-(4-*n*-hexyloxyphenyl)-4,2′:6′,4″-tpy reacts with Co(NCS)_2_; a combination of 4′-(4-*n*-nonyloxyphenyl)-4,2′:6′,4″-tpy and Co(NCS)_2_ also gives rise to an analogous 3D-net [24]. In [{Co(**2**)_2_(NCS)_2_}**^.^**0.6CHCl_3_]*_n_*, [{Co(**3**)_2_(NCS)_2_}**^.^**4CHCl_3_**^.^**0.25H_2_O]*_n_* and [{Co(**4**)_2_(NCS)_2_}**^.^**4CHCl_3_]*_n_*, the 3,2′:6′,3″-tpy domains all adopt the conformation depicted in Scheme 5a. In contrast, on going to the ligands with the *n*-pentyloxy and *n*-hexyloxy chains, the conformation switches to that shown in Scheme 5b. The angles between the least squares planes of the pyridine rings in the 3,2′:6′,3″-tpy unit also change (Table 2). These changes are associated with a modification of the rhombuses that make up the (4,4) net, as discussed below. 

**Figure 4:**
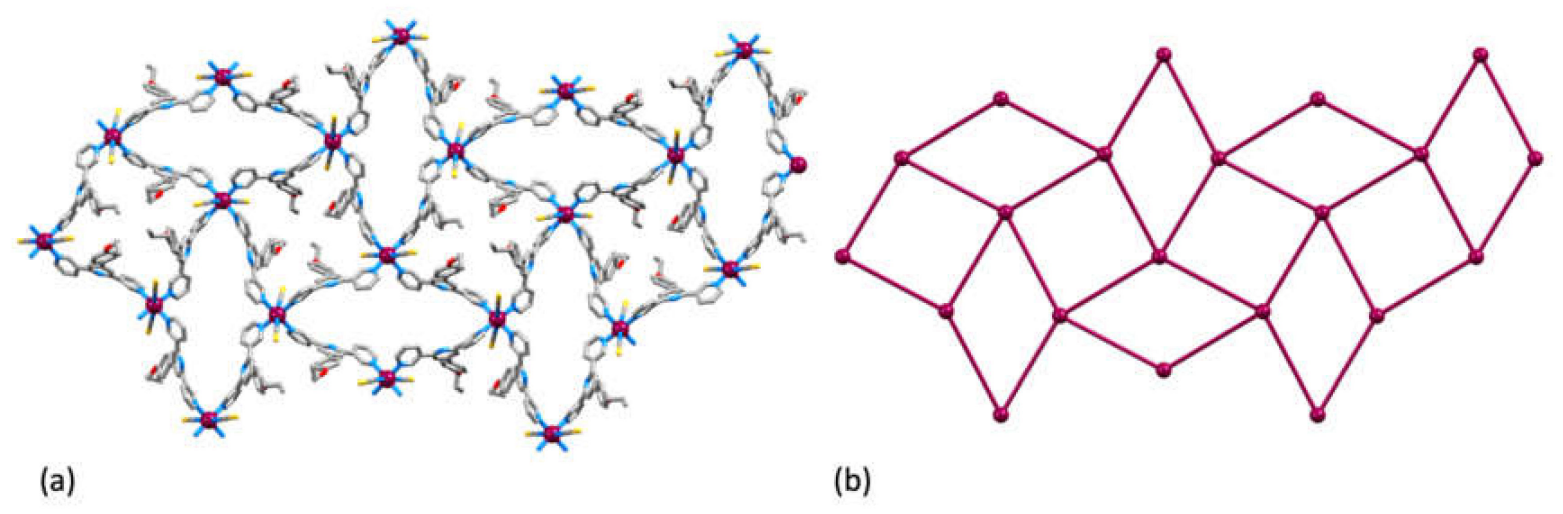
(**a**) Part of one (4,4) net (viewed down the crystallographic *c*-axis) in [{Co(**4**)_2_(NCS)_2_}**^.^**4CHCl_3_]*_n_*; H atoms and solvent molecules are omitted. (**b**) The same net defined by lines connecting the Co atoms.

**Figure 5:**
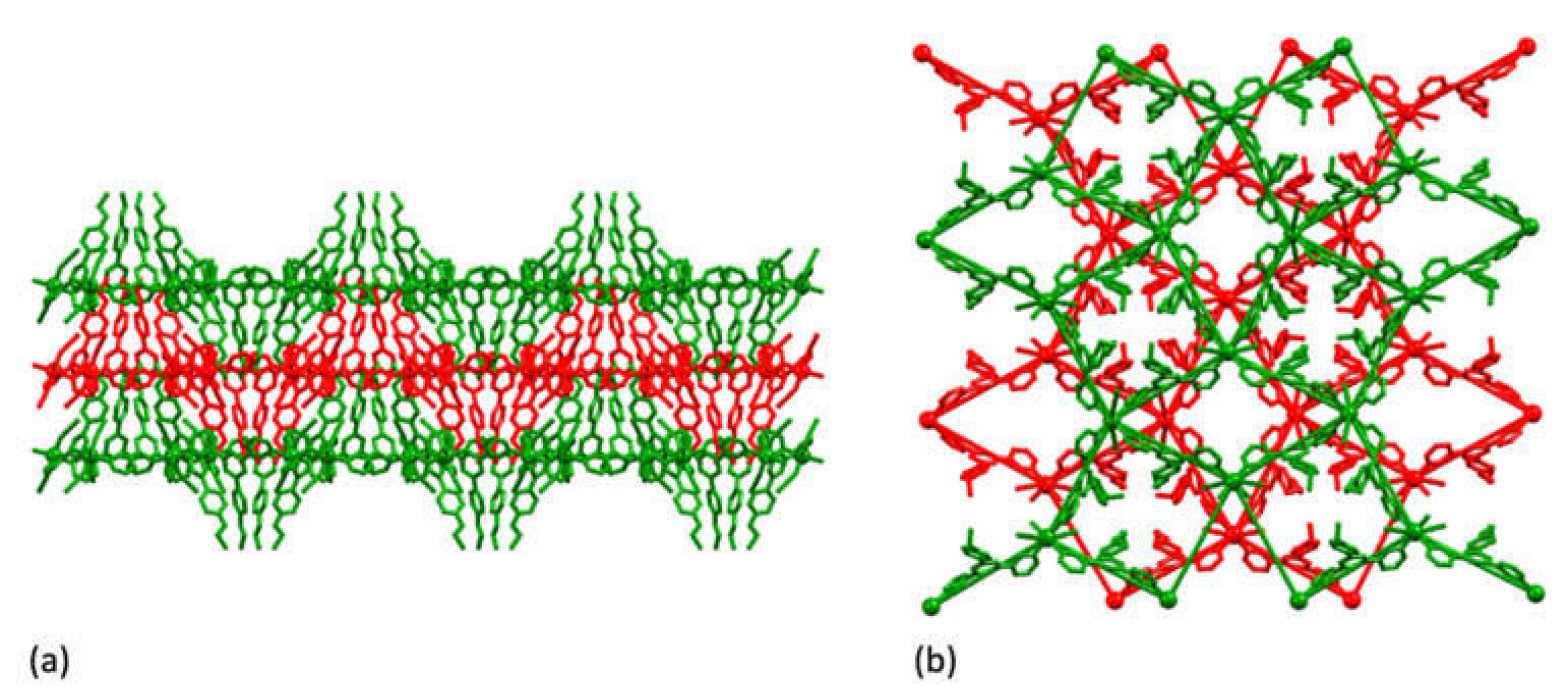
(**a**) Packing of (4,4) nets (viewed down the crystallographic *a*-axis) in [{Co(**2**)_2_(NCS)_2_}**^.^**0.6CHCl_3_]*_n_*. (**b**) Packing of (4,4) nets (viewed down the crystallographic *c*-axis) in [{Co(**3**)_2_(NCS)_2_}**^.^**4CHCl_3_**^.^**0.25H_2_O]*_n_*, with each net defined by the lines connecting the Co atoms. Hydrogen atoms and solvent molecules are omitted.

We consider first the (4,4) nets assembled with Co(NCS)_2_ and ligands **2**–**4**. The net is geometrically the same in [{Co(**2**)_2_(NCS)_2_}**^.^**0.6CHCl_3_]*_n_*, [{Co(**3**)_2_(NCS)_2_}**^.^**4CHCl_3_**^.^**0.25H_2_O]*_n_* and [{Co(**4**)_2_(NCS)_2_}**^.^**4CHCl_3_]*_n_*, and is shown in Figure 4. Each net comprises two geometrically distinct rhombuses with Co...Co separations in the range of 13.224 to 13.470(1) Å. In [{Co(**2**)_2_(NCS)_2_}**^.^**0.6CHCl_3_]*_n_*, the Co atoms are positioned along a 2-fold axis, and all Co...Co separations are 13.224 Å. Working around the circuit of each square (or near-square in the cases of [{Co(**3**)_2_(NCS)_2_}**^.^**4CHCl_3_**^.^**0.25H_2_O]*_n_* and [{Co(**4**)_2_(NCS)_2_}**^.^**4CHCl_3_]*_n_*) motif, the four 4-*n*-alkyloxyphenyl substituents all point in the same direction, and for adjacent corner-sharing squares sets of four *n*-alkyloxy chains point alternately up and down (Figure 4a and Figure S16 in the supporting information). This leads to a cone-like arrangement, and adjacent sheets pack with the cones nesting into one another (Figure 5a). The sheets pack in an ABAB... manner with adjacent sheets twisted through 90° with respect to the next (Figure 5b). The transition from an ethoxy to *n*-propoxy to *n*-butoxy substituent has little impact on the structure, with the distances between the mean planes that are constructed through the cobalt atoms in a sheet increasing only from 9.07 Å in [{Co(**2**)_2_(NCS)_2_}**^.^**0.6CHCl_3_]*_n_* to 9.56 Å in [{Co(**4**)_2_(NCS)_2_}**^.^**4CHCl_3_]*_n_*. We note that π-stacking interactions play no role in the assembly.

**Figure 6:**
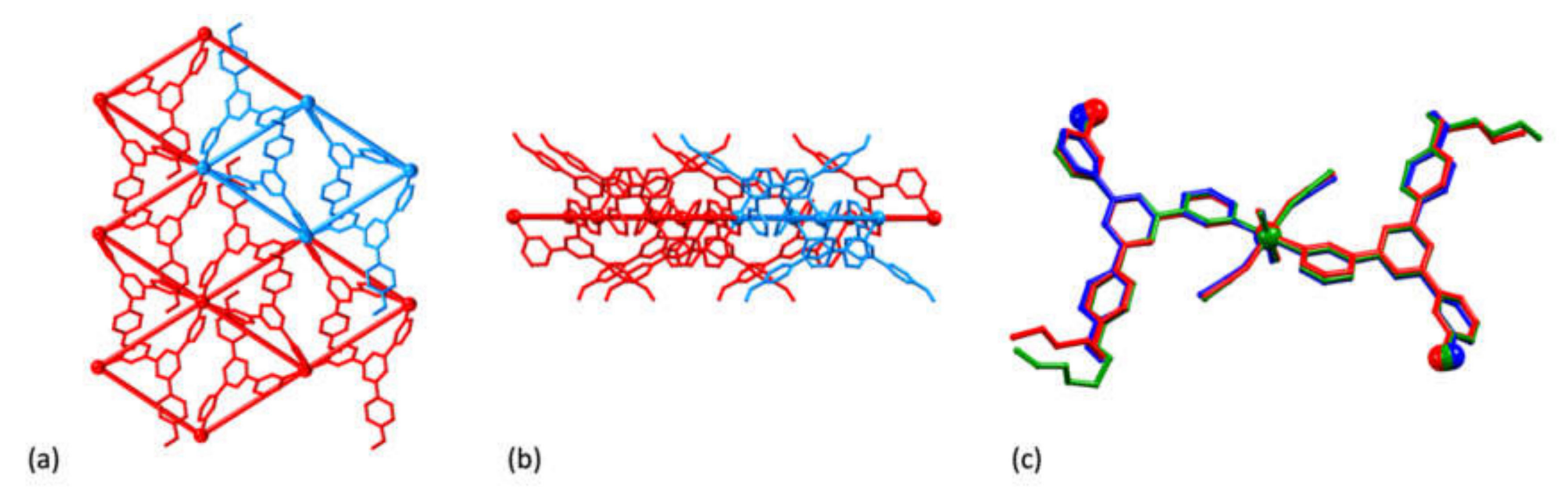
Part of one (4,4) net in [{Co(**1**)_2_(NCS)_2_}**^.^**3MeOH]*_n_* with the net defined by the lines connecting the Co atoms viewed (**a**) from above and (**b**) into the plane containing the Co atoms; H atoms, thiocyanato ligands and solvent molecules are omitted. One rhombus with the four ligands **1** that link Co centres is highlighted in blue. CSD refcode FOXQUH [19]. (**c**) Overlay of the repeat units with symmetry-generated atoms in [{Co(**1**)_2_(NCS)_2_}**^.^**3MeOH]*_n_* (in blue), [Co_2_(**5**)_4_(NCS)_4_]*_n_* (in red) and [Co(**6**)_2_(NCS)_2_]*_n_* (in green).

**Figure 7:**
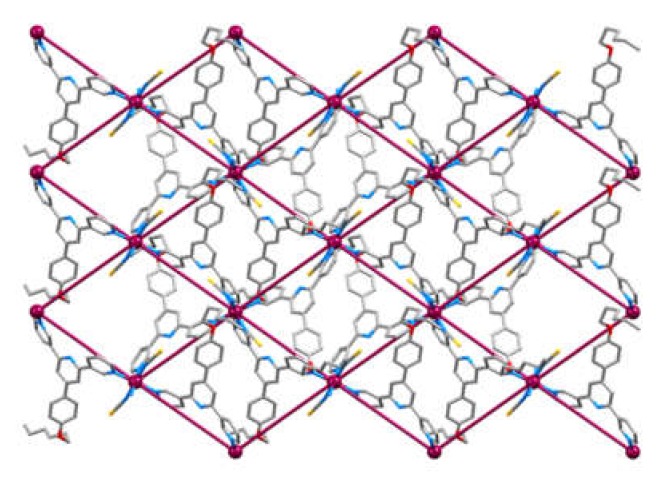
Part of one (4,4) net in [Co(**6**)_2_(NCS)_2_]*_n_* with the net defined by the lines connecting the Co atoms; H atoms are omitted.

**Figure 8:**
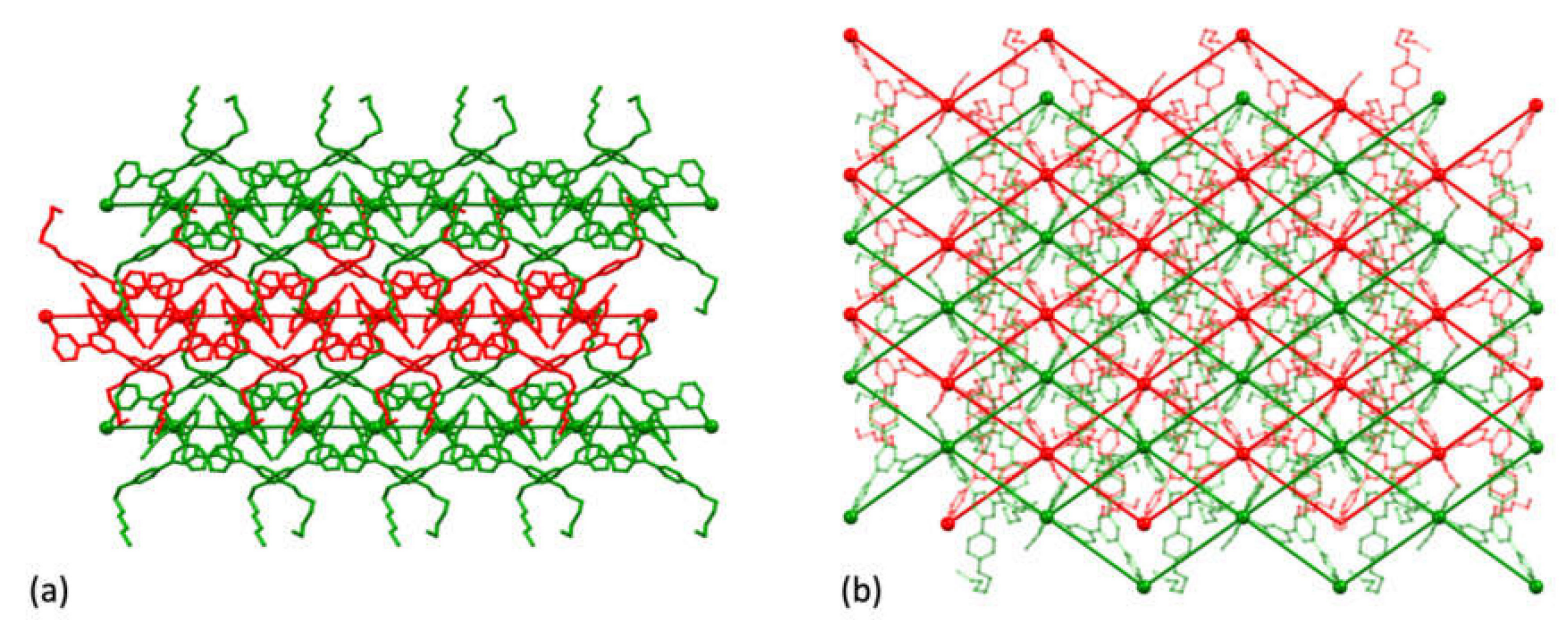
Staggered 2D-sheets in [Co(**6**)_2_(NCS)_2_]*_n_*: (**a**) view down the *a-*axis, and (**b**) view down the *c*-axis. H atoms are omitted. An analogous packing of sheets occurs in [Co_2_(**5**)_4_(NCS)_4_]*_n_*.

The network common to the compounds with ligands **2**, **3** and **4** differs from that observed for the analogous compound containing ligand **1** with a methoxy substituent [19]. In the pseudopolymorphs [{Co(**1**)_2_(NCS)_2_}**^.^**2.2CHCl_3_]*_n_* and [{Co(**1**)_2_(NCS)_2_}**^.^**3MeOH]*_n_,* ligand **1** adopts the conformation shown in Scheme 5b, and the network comprises identical rhombuses with the ligands pointing above and below a rhombus (Figure 6a,b). Rather unexpectedly, we observe that ongoing from ligands **2**–**4** to ligands **5** and **6**, each (4,4) net in [Co_2_(**5**)_4_(NCS)_4_]*_n_* and [Co(**6**)_2_(NCS)_2_]*_n_* reverts to the assembly pattern observed for ligand **1**. Figure 6c shows an overlay of the repeat units with symmetry-generated atoms in [{Co(**1**)_2_(NCS)_2_}**^.^**3MeOH]*_n_* (CSD refcode FOXQUH [19]), [Co_2_(**5**)_4_(NCS)_4_]*_n_* and [Co(**6**)_2_(NCS)_2_]*_n_*, revealing the similarities in the structural motifs. The (4,4) nets in [Co(**6**)_2_(NCS)_2_]*_n_* and [Co_2_(**5**)_4_(NCS)_4_]*_n_* are displayed in Figure 7 and Figure S18, respectively, and a comparison of these figures with Figure 6a is instructive. However, the packing of the sheets is significantly affected by the introduction of the longer alkyloxy chains. In [{Co(**1**)_2_(NCS)_2_}**^.^**3MeOH]*_n_*, the sheets are aligned with eclipsed rhombuses (Figure S19). In contrast, in [Co_2_(**5**)_4_(NCS)_4_]*_n_* and [Co(**6**)_2_(NCS)_2_]*_n_*, the 2D-networks pack in an ABAB... fashion (Figure 8), allowing the *n-*pentyloxy and *n-*hexyloxy to penetrate into the cavities in an adjacent sheet.

A significant point is that in the case of the reaction of Co(NCS)_2_ with ligand **1**, there is competition between the formation of a (4,4) net ([{Co(**1**)_2_(NCS)_2_}**^.^**2.2CHCl_3_]*_n_* or [{Co(**1**)_2_(NCS)_2_}**^.^**3MeOH]*_n_* [19]) and a 1D-coordination polymer [Co(**1**)(NCS)_2_(MeOH)_2_]*_n_*, with the 1D-coordination polymer being the dominant product [19]. Powder XRD confirmed that this is not the case for the coordination polymers formed with ligands **5** and **6**, and the single-crystal structures of [Co_2_(**5**)_4_(NCS)_4_]*_n_* and [Co(**6**)_2_(NCS)_2_]*_n_* are representative of the bulk materials. While the details of a single 2D-net are the same in each of [{Co(**1**)_2_(NCS)_2_}**^.^**2.2CHCl_3_]*_n_*, [{Co(**1**)_2_(NCS)_2_}**^.^**3MeOH]*_n_*, [Co_2_(**5**)_4_(NCS)_4_]*_n_* and [Co(**6**)_2_(NCS)_2_]*_n_*, our observations suggest that longer *n*-alkyloxy chains and their penetration through voids in an adjacent layer are contributing factors to the preference for a 2D-network rather than a 1D-polymer. Another important point is that [Co_2_(**5**)_4_(NCS)_4_]*_n_* and [Co(**6**)_2_(NCS)_2_]*_n_* crystallize without a lattice solvent, consistent with the efficient packing of the 2D-nets. In contrast, lattice solvent plays an important role in the pseudopolymorphs [{Co(**1**)_2_(NCS)_2_}**^.^**3MeOH]*_n_* and [{Co(**1**)_2_(NCS)_2_}**^.^**2.2CHCl_3_]*_n_* [19].

## 3. Materials and Methods 

### 3.1. General

^1^H and ^13^C NMR spectra were recorded on a Bruker Avance iii-500 spectrometer (Bruker BioSpin AG, Fällanden, Switzerland) at 298 K. The ^1^H and ^13^C NMR chemical shifts were referenced with respect to residual solvent peaks (*δ* TMS = 0). A Shimadzu LCMS-2020 and a Bruker maXis 4G QTOF instrument (Bruker BioSpin AG, Fällanden, Switzerland) were used to record electrospray ionization (ESI) and HR-ESI mass spectra, respectively. PerkinElmer UATR Two (Perkin Elmer, 8603 Schwerzenbach, Switzerland) and Shimadzu UV2600 (Shimadzu Schweiz GmbH, 4153 Reinach, Switzerland) instruments were used to record FT-infrared (IR) and absorption spectra, respectively.

3-Acetylpyridine, 4-hydroxybenzaldehyde and 1-bromopropane were purchased from Acros Organics (Chemie Brunschwig AG, Basel, Switzerland) and were used as received. Ligands **2**, **4**, **5** and **6** were prepared as previously reported [11,20,21].

### 3.2. Compound **3**

4-Hydroxybenzaldehyde (3.05 g, 25.0 mmol) was dissolved in EtOH (50 mL), then 3-acetylpyridine (6.06 g, 5.51 mL, 50.0 mmol) and crushed KOH (2.81 g, 50.0 mmol) were added to the solution. Aqueous NH_3_ (32%, 80 mL) was slowly added to the reaction mixture. This was stirred at room temperature overnight. The excess of NH_3_ was removed under vacuum, then water (100 mL) was added to the reaction mixture and CH_2_Cl_2_ was used to extract impurities. Aqueous HCl (ca. 4%) was added to the aqueous phase until the pH = 8 and the formation of a solid was observed. The yellow solid was collected by filtration, washed with water (3 × 10 mL) and dried in vacuo. The ^1^H–NMR spectrum of the product was in agreement with that previously reported for 4′-(4-hydroxyphenyl)-3,2′:6′,3″-terpyridine (3.10 g, 9.53 mmol, 38.1%) [22]. All the intermediate (3.10 g, 9.53 mmol) was dissolved in DMF (40 mL), and the solution was heated to 70 °C. Then, anhydrous K_2_CO_3_ (3.95 g, 28.6 mmol) was added, and the color changed from yellow to red-brown. After 5 min, 1-bromopropane (1.29 g, 0.96 mL, 10.5 mmol) was added to the reaction mixture. This was stirred at 70 °C overnight, then it was cooled to room temperature and poured into ice water and stirred for 20 min. The brown solid that formed was dissolved in CHCl_3_ and was washed with aqueous K_2_CO_3_ solution. The organic layers were dried over MgSO_4_, and the solvent was then removed. The solid product was purified by column chromatography (SiO_2_, ethylacetate:cyclohexane 1:1, R_f_ = 0.16). Compound **3** was obtained as a pale yellow solid (1.32 g, 3.60 mmol, 37.8%). M.p. = 83 °C. ^1^H–NMR (500 MHz, CDCl_3_): *δ*/ppm 9.37 (d, *J* = 0.8 Hz, 2H, H^A2^), 8.70 (dd, *J* = 4.8, 1.7 Hz, 2H, H^A6^), 8.50 (dt, *J* = 7.9, 2.0 Hz, 2H, H^A4^), 7.92 (s, 2H, H^B3^), 7.71 (m, 2H, H^C2^), 7.46 (m, 2H, H^A5^), 7.07 (m, 2H, H^C3^), 4.01 (t, *J* = 6.6 Hz, 2H, H^a^), 1.86 (m, 2H, H^b^), 1.08 (t, *J* = 7.4 Hz, 3H, H^c^). ^13^C{^1^H}–NMR (500 MHz, CDCl_3_): *δ*/ppm 160.6 (C^C4^), 155.4 (C^A3^), 150.6 (C^B4^), 150.2 (C^A6^), 148.5 (C^A2^), 135.0 (C^B2^), 134.7 (C^A4^), 130.3 (C^C1^), 128.5 (C^C2^), 123.8 (C^A5^), 117.3 (C^B3^), 115.4 (C^C3^), 69.9 (C^a^), 22.7 (C^b^), 10.7 (C^c^). UV-VIS (CH_3_CN, 2.0 × 10^−5^ mol dm^−3^) λ/nm 227 (ε/dm^3^ mol^−1^ cm^−1^ 26,500), 275 (31,700). ESI-MS *m/z* 368.15 [M+H]^+^ (calc. 368.18). HR-MS *m/z* 368.1764 [M+H]^+^ (calc. 368.1757). IR (selected bands, see Figure S6 in the supporting information)/cm^−1^: 3041w, 2962w, 2934w, 1603vs, 1515vs, 1243vs, 1182s, 1024s, 804vs, 700vs.

### 3.3. Crystal Growth of [{Co(**2**)_2_(NCS)_2_}**^.^**0.6CHCl_3_]_n_

In experiment I, a solution of Co(NCS)_2_ (5.3 mg, 0.030 mmol) in MeOH (6 mL) was layered over a CHCl_3_ solution (4 mL) of **2** (10.6 mg, 0.030 mmol) in a crystallization tube (i.d. = 13.6 mm, 24 mL). Pink block-like crystals grew after four days (total mass of product was ca. 10 mg), and a single crystal was selected for X-ray diffraction. The single-crystal structural data confirmed a formulation of [{Co(**2**)_2_(NCS)_2_}**^.^**0.6CHCl_3_]*_n_*.

In experiment II, a solution of Co(NCS)_2_ (10.6 mg, 0.060 mmol) in MeOH (6 mL) was layered over a CHCl_3_ solution (4 mL) of **2** (10.6 mg, 0.030 mmol) in a crystallization tube (i.d. = 13.6 mm, 24 mL). Pink block-like crystals grew after four days (total mass of product was ca. 10 mg), and a single crystal was selected. A cell check confirmed a match with the crystal structurally characterized from experiment I. The remaining crystals in the tube were washed with MeOH and CHCl_3_, dried under vacuum and analyzed by PXRD and FT-IR spectroscopy. IR (selected bands, see Figure S7 in the supporting information)/cm^−1^: 2975w, 2068vs, 1601vs, 1514vs, 1242vs, 1179s, 1050s, 815s, 747vs, 703vs.

In experiment III, a solution of Co(NCS)_2_ (2.7 mg, 0.015 mmol) in MeOH (6 mL) was layered over a CHCl_3_ solution (4 mL) of **2** (10.6 mg, 0.030 mmol) in a crystallization tube (i.d. = 13.6 mm, 24 mL). Pink block-like crystals grew after five days (total mass of product was ca. 10 mg), and a single crystal was selected for X-ray diffraction. A cell check confirmed a match with the crystal structurally characterized from experiment I.

### 3.4. Crystal Growth of [{Co(**3**)_2_(NCS)_2_}**^.^**4CHCl_3_**^.^**0.25H_2_O]_n_

In experiment I, a solution of Co(NCS)_2_ (5.3 mg, 0.030 mmol) in MeOH (5 mL) was layered over a CHCl_3_ solution (4 mL) of **3** (11.0 mg, 0.030 mmol) in a crystallization tube (i.d. = 13.6 mm, 24 mL). Pink block-like crystals grew after one day (total mass of product was ca. 10 mg), and a single crystal was selected for X-ray diffraction. The structural data confirmed a formulation of [{Co(**3**)_2_(NCS)_2_}**^.^**4CHCl_3_**^.^**0.25H_2_O]*_n_*.

In experiment II, a solution of Co(NCS)_2_ (10.6 mg, 0.060 mmol) in MeOH (5 mL) was layered over a CHCl_3_ solution (4 mL) of **3** (11.0 mg, 0.030 mmol) in a crystallization tube (i.d. = 13.6 mm, 24 mL). Pink block-like crystals grew after one day, and a single crystal was selected for X-ray diffraction. A cell check confirmed a match with the crystal structurally characterized from experiment I. The remaining crystals in the tube were washed with MeOH and CHCl_3_, dried under vacuum and analyzed by PXRD and FT-IR spectroscopy. IR (selected bands, see Figure S8 in the supporting information)/cm^−1^: 2968w, 2068vs, 1602vs, 1514vs, 1241vs, 1179s, 977m, 816s, 748vs, 704vs.

### 3.5. Crystal Growth of [{Co(**4**)_2_(NCS)_2_}**^.^**4CHCl_3_]_n_

In experiment I, a solution of Co(NCS)_2_ (5.3 mg, 0.030 mmol) in MeOH (6 mL) was layered over a CHCl_3_ solution (4 mL) of **4** (11.4 mg, 0.030 mmol) in a crystallization tube (i.d. = 13.6 mm, 24 mL). Pink plate-like crystals grew after five days (total mass of product was ca. 10 mg), and a single crystal was selected for X-ray diffraction. The structural data confirmed a formulation of [{Co(**4**)_2_(NCS)_2_}**^.^**4CHCl_3_]*_n_*.

In experiment II, a solution of Co(NCS)_2_ (10.6 mg, 0.060 mmol) in MeOH (6 mL) was layered over a CHCl_3_ solution (4 mL) of **4** (11.4 mg, 0.030 mmol) in a crystallization tube (i.d. = 13.6 mm, vol. = 24 mL). Pink plate-like crystals grew after four days, and a single crystal was selected for X-ray diffraction. A cell check confirmed a match with the crystal structurally characterized from experiment I. The remaining crystals in the tube were washed with MeOH and CHCl_3_, dried under vacuum and analyzed by PXRD and FT-IR spectroscopy. IR (selected bands, see Figure S9 in the supporting information)/cm^−1^: 2958w, 2062vs, 1600vs, 1514vs, 1240vs, 1180s, 1032m, 815s, 748vs, 703vs.

### 3.6. Crystal Growth of [Co_2_(**5**)_4_(NCS)_4_]_n_

A solution of Co(NCS)_2_ (10.6 mg, 0.060 mmol) in MeOH (6 mL) was layered over a CHCl_3_ solution (4 mL) of **5** (11.9 mg, 0.030 mmol) in a crystallization tube (i.d. = 13.6 mm, 24 mL). Pink plate-like crystals grew after three months (total mass of product was ca. 5 mg), and a single crystal was selected for X-ray diffraction. The structural data confirmed a formulation of [Co_2_(**5**)_4_(NCS)_4_]*_n_*. The remaining crystals in the tube were washed with MeOH and CHCl_3_, dried under vacuum and analyzed by PXRD and FT-IR spectroscopy. IR (selected bands, see Figure S10 in the supporting information)/cm^−1^: 2929w, 2074vs, 1607s, 1510s, 1226s, 1172s, 1049s, 811vs, 703vs.

### 3.7. Crystal Growth of [Co(**6**)_2_(NCS)_2_]_n_

A solution of Co(NCS)_2_ (5.3 mg, 0.030 mmol) in MeOH (6 mL) was layered over a CH_2_Cl_2_ solution (4 mL) of **6** (12.3 mg, 0.030 mmol) in a crystallization tube (i.d. = 13.6 mm, 24 mL). Pink plate-like crystals grew after six days (total mass of product was < 10 mg), and a single crystal was selected for X-ray diffraction. The structural data confirmed a formulation of [Co(**6**)_2_(NCS)_2_]*_n_*. The remaining crystals in the tube were washed with MeOH and CH_2_Cl_2_, dried under vacuum and analyzed by PXRD and FT-IR spectroscopy. IR (selected bands, see Figure S11 in the supporting information)/cm^−1^: 2927w, 2072vs, 1606vs, 1512vs, 1247vs, 1175vs, 1032m, 807vs, 702vs.

### 3.8. Crystallography

Single crystal data were collected on a Bruker APEX-II diffractometer (CuKα radiation) with data reduction, solution and refinement using the programs APEX [25], ShelXT [26], Olex2 [27] and ShelXL v. 2014/7 [28], and/or using a STOE StadiVari diffractometer equipped with a Pilatus300K detector and with a Metaljet D2 source (GaKα radiation) and solving the structure using Superflip [29,30] and Olex2 [27]; the model was refined with ShelXL v. 2014/7 [28]. The structure analysis, including the ORTEP representations, used Mercury CSD v. 4.1.0 [31,32].

Powder X-Ray Diffraction (PXRD) patterns were collected at room temperature in transmission mode using a Stoe Stadi P diffractometer equipped with a Cu Kα1 radiation (Ge(111) monochromator) and a DECTRIS MYTHEN 1K detector. The reflections of the bulk samples of [{Co(**2**)_2_(NCS)_2_}**^.^**0.6CHCl_3_]*_n_*, [{Co(**3**)_2_(NCS)_2_}**^.^**4CHCl_3_**^.^**0.25H_2_O]*_n_* and [{Co(**4**)_2_(NCS)_2_}**^.^**4CHCl_3_]*_n_* were indexed with the tetragonal cells *P*4*/ncc*, *P–*42_1_*c* and *P–*42_1_*c*, respectively. The reflections of the bulk samples of [Co_2_(**5**)_4_(NCS)_4_]*_n_* and [Co(**6**)_2_(NCS)_2_]*_n_* were indexed with the monoclinic cells *P2_1_/n* and *P*2_1_*/c*, respectively. The profile matching analysis [33,34,35] of the diffraction patterns was performed with the package FULLPROF SUITE [35,36] (version July-2019) using a previously determined instrument resolution function based on a NIST640d standard. The structural models were taken from the single crystal X-Ray diffraction refinements. The refined parameters in Profile matching were: zero shift, lattice parameters, peak asymmetry, sample transparency and peak shapes as a Thompson-Cox-Hastings pseudo-Voigt function. The refinements confirmed that the bulk samples were consistent with the single crystal structures for all the compounds.

### 3.9. [{Co(**2**)_2_(NCS)_2_}**^.^**0.6CHCl_3_]_n_

C_50.40_H_40.40_Cl_7.20_CoN_8_O_2_S_2_, *M*_r_ = 1168.39, pink block, tetragonal, space group *P*4*/ncc*, *a* = *b* = 25.3093(5), *c* = 18.1347(5) Å, *V* = 11616.4(6) Å^3^, *D*_c_ = 1.336 g cm^−3^, *T* = 130 °K, *Z* = 8, *Z’* = 0.5, μ(Ga*Kα*) = 4.267 mm^−1^. Total 81,455 reflections, 6020 unique (*R_int_* = 0.1459). Refinement of 3155 reflections (330 parameters) with *I* > 2*σ*(*I*) converged at final *R*_1_ = 0.0963 (*R*_1_ all data = 0.1675), *wR*_2_ = 0.2722 (*wR*2 all data = 0.3398), gof = 1.028. CCDC 1982103. 

### 3.10. [{Co(**3**)_2_(NCS)_2_}**^.^**4CHCl_3_**^.^**0.25H_2_O]_n_

C_54_H_46.50_Cl_12_CoN_8_O_2.25_S_2_, *M*_r_ = 1391.94, pink block, tetragonal, space group *P–*42_1_*c*, *a* = *b* = 25.6618(13), *c* = 18.709(1) Å, *V* = 12320.4(14) Å^3^, *D*_c_ = 1.501 g cm^−3^, *T* = 130 °K, *Z* = 8, *Z’* = 1, μ(Cu*Kα*) = 7.998 mm^−1^. Total 68,222 reflections, 11,345 unique (*R_int_* = 0.0469). Refinement of 10,789 reflections (720 parameters) with *I* > 2*σ*(*I*) converged at final *R*_1_ = 0.0684 (*R*_1_ all data = 0.0712), *wR*_2_ = 0.1962 (*wR*2 all data = 0.1998), gof = 1.115. CCDC 1982101.

### 3.11. [{Co(**4**)_2_(NCS)_2_}**^.^**4CHCl_3_]_n_

C_56_H_50_Cl_12_CoN_8_O_2_S_2_, *M*_r_ = 1417.50, pink plate, tetragonal, space group *P–*42_1_*c*, *a* = *b* = 25.7185(3), *c* = 19.1253(3) Å, *V* = 12650.3(4) Å^3^, *D*_c_ = 1.489 g cm^−3^, *T* = 130 °K, *Z* = 8, *Z’* = 1, μ(Ga*Kα*) = 5.173 mm^−1^. Total 87,950 reflections, 12,678 unique (*R_int_* = 0.0925). Refinement of 9999 reflections (733 parameters) with *I* > 2*σ*(*I*) converged at final *R*_1_ = 0.0626 (*R*_1_ all data = 0.0789), *wR*_2_ = 0.1655 (*wR*2 all data = 0.1710), gof = 1.030. CCDC 1982105.

### 3.12. [Co_2_(**5**)_4_(NCS)_4_]_n_

C_108_H_100_Co_2_N_16_O_4_S_4_, *M*_r_ = 1932.13, pink plate, monoclinic, space group *P2_1_/n*, *a* = 13.1357(4), *b* = 12.4666(3), *c* = 15.1441(4) Å, *β* = 100.148(2)°, *V* = 2441.16(12) Å^3^, *D*_c_ = 1.314 g cm^−3^, *T* = 130 °K, *Z* = 1, *Z’* = 0.25, μ(Ga*Kα*) = 2.675 mm^−1^. Total 36,315 reflections, 4983 unique (*R_int_* = 0.0621). Refinement of 4596 reflections (305 parameters) with *I* > 2*σ*(*I*) converged at final *R*_1_ = 0.0500 (*R*_1_ all data = 0.0555), *wR*_2_ = 0.1376 (*wR*2 all data = 0.1453), gof = 1.113. CCDC 1982104. 

### 3.13. [Co(**6**)_2_(NCS)_2_]_n_

C_56_H_54_CoN_8_O_2_S_2_, *M*_r_ = 994.12, pink plate, monoclinic, space group *P*2_1_*/c*, *a* = 18.3988(14), *b* = 12.9419(10), *c* = 21.7046(17) Å, *β* = 98.421(4)^o^, *V* = 5112.5(7) Å^3^, *D*_c_ = 1.292 g cm^−3^, *T* = 130 °K, *Z* = 4, *Z’* = 1, μ(Cu*Kα*) = 3.787 mm^−1^. Total 61,349 reflections, 9388 unique (*R_int_* = 0.0353). Refinement of 8264 reflections (624 parameters) with *I* > 2*σ*(*I*) converged at final *R*_1_ = 0.0467 (*R*_1_ all data = 0.0534), *wR*_2_ = 0.1241 (*wR*2 all data = 0.1298), gof = 1.047. CCDC 1982102.

## 4. Conclusions

We have investigated the assembly of five 2D-coordination networks from reactions of Co(NCS)_2_ with 4′-(4-*n*-alkyloxyphenyl)-3,2′:6′,3″-terpyridines, in which the *n*-alkyl group is ethyl, *n*-propyl, *n*-butyl, *n*-pentyl and *n*-hexyl in ligands **2**–**6**, respectively. Each of [{Co(**2**)_2_(NCS)_2_}**^.^**0.6CHCl_3_]*_n_*, [{Co(**3**)_2_(NCS)_2_}**^.^**4CHCl_3_**^.^**0.25H_2_O]*_n_*, [{Co(**4**)_2_(NCS)_2_}**^.^**4CHCl_3_]*_n_*, [Co_2_(**5**)_4_(NCS)_4_]*_n_* and [Co(**6**)_2_(NCS)_2_]*_n_* exhibits a (4,4) net with cobalt atoms as 4-connecting nodes. For the compounds with ligands **2**, **3** and **4**, the net contains two geometrically different rhombuses, and the nets pack in an ABAB... arrangement with sets of four pendant *n*-alkyloxyphenyl groups forming a cone-like array which nests into an analogous unit in the next sheet. No π-stacking interactions are observed. Ongoing from the networks with ligands **2**–**4** to [Co_2_(**5**)_4_(NCS)_4_]*_n_* and [Co(**6**)_2_(NCS)_2_]*_n_*, in which the ligands bear *n*-pentyloxy and *n*-hexyloxy chains, the conformation of the 3,2′:6′,3″-tpy unit changes (Scheme 5) and redirects the (4,4) net to one in which the rhombuses are all identical. Although this mimics a single net in [{Co(**1**)_2_(NCS)_2_}**^.^**3MeOH]*_n_* and [{Co(**1**)_2_(NCS)_2_}**^.^**2.2CHCl_3_]*_n_* [19], the change from a peripheral methoxy substituent in **1** to the longer chains in **5** and **6** modifies the arrangement of adjacent sheets. In [Co_2_(**5**)_4_(NCS)_4_]*_n_* and [Co(**6**)_2_(NCS)_2_]*_n_*, the (4,4) nets pack in an ABAB... manner, allowing the *n*-alkyloxy tails to be accommodated in cavities in an adjacent layer. The results have demonstrated that a combination of a variable chain length of the 4′-(4-*n*-alkyloxyphenyl) substituents and a conformationally flexible 3,2′:6′,3″-tpy metal-binding domain redirects the coordination network assembly while retaining a (4,4) net.

## Figures and Tables

**Table 1:** Bond lengths and angles in the cobalt(II) coordination sphere.

Compound	Co–N_tpy_/Å	Co–N_NCS_/Å	N_tpy_–Co–N_tpy_^1^/deg	N_NCS_–Co–N_NCS_/deg
[{Co(**2**)_2_(NCS)_2_}**^.^**0.6CHCl_3_]*_n_*	2.172(5), 2.159(5)	2.083(7)	174.4(2)	178.0(3)
[{Co(**3**)_2_(NCS)_2_}**^.^**4CHCl_3_**^.^**0.25H_2_O]*_n_* ^2^	2.211(6), 2.200(5), 2.183(6), 2.164(6)	2.067(7), 2.084(7)	175.5(2), 174.0(2)	177.0(2)
[{Co(**4**)_2_(NCS)_2_}**^.^**4CHCl_3_]*_n_* ^2^	2.159(5), 2.172(5), 2.215(5), 2.229(5)	2.075(6), 2.083(6)	175.8(2), 173.78(19)	177.5(2)
[Co_2_(**5**)_4_(NCS)_4_]*_n_*	2.1693(18), 2.2175(18)	2.0870(19)	180.00(9), 180.0	180.0
[Co(**6**)_2_(NCS)_2_]*_n_* ^2^	2.1857(19), 2.1789(19), 2.2237(18), 2.2356(18)	2.0754(18), 2.0799(18)	179.22(7), 178.59(7)	179.24(7)

^1^ Only the *trans* angles are given. ^2^ Two independent ligands.

**Table 2:** Angles between the least squares planes of the rings in the 3,2′:6′,3″-tpy units.

Compound	angle 1^1^/deg	angle 2^1^/deg
[{Co(**2**)_2_(NCS)_2_}**^.^**0.6CHCl_3_]*_n_*	28.1	43.9
[{Co(**3**)_2_(NCS)_2_}**^.^**4CHCl_3_**^.^**0.25H_2_O]*_n_* ^2^	22.6, 26.1	40.5, 40.6
[{Co(**4**)_2_(NCS)_2_}**^.^**4CHCl_3_]*_n_* ^2^	28.5, 23.1	37.9, 38.1
[Co_2_(**5**)_4_(NCS)_4_]*_n_*	28.5	32.8
[Co(**6**)_2_(NCS)_2_]*_n_* ^2^	29.7, 29.9	30.8, 33.3

^1^ For the conformation in Scheme 5b, angles 1 and 2 are defined for the left- and right-hand pairs of rings, respectively. ^2^ Two independent ligands.

## Supplementary Materials

The following are available online at https://www.mdpi.com/1420-3049/25/7/1663/s1, Figures S1–S6: Mass spectra, NMR and IR spectra of compound **3**; Figures S7–S11: solid-state IR spectra of the five coordination networks; Figures S12–S16: ORTEP plots; Figures S17–S19: additional structural and packing diagrams.

## References

[1] Constable E.C. (2007). 2,2′:6′,2″-Terpyridines: From chemical obscurity to common supramolecular motifs. Chem. Soc. Rev..

[2] Wild A., Winter A., Schlütter F., Schubert U.S. (2011). Advances in the field of π-conjugated 2,2′:6′,2″-terpyridines. Chem. Soc. Rev..

[3] Wei C., He Y., Shi X., Song Z. (2019). Terpyridine-metal complexes: Applications in catalysis and supramolecular chemistry. Coord. Chem. Rev..

[4] Agosti A., Kuna E., Bergamini G. (2019). Divergent terpyridine-based coordination for the construction of photoactive supermolecular structures. Eur. J. Inorg. Chem..

[5] Chakraborty S., Newkome G.R. (2018). Terpyridine-based metallosupramolecular constructs: Tailored monomers to precise 2D-motifs and 3D-metallocages. Chem. Soc. Rev..

[6] Constable E.C., Housecroft C.E. (2017). More Hydra than Janus*—*Non-classical coordination modes in complexes of oligopyridine ligands. Coord. Chem. Rev..

[7] Kröhnke F. (1976). The specific synthesis of pyridines and oligopyridines. Synthesis.

[8] Wang J., Hanan G.S. (2005). A facile route to sterically hindered and non-hindered 4′-aryl-2,2′:6′,2″-terpyridines. Synlett.

[9] Groom C.R., Bruno I.J., Lightfoot M.P., Ward S.C. (2016). The cambridge structural database. Acta Cryst..

[10] Housecroft C.E. (2015). Divergent 4,2′:6′,4′′- and 3,2′:6′,3′′-Terpyridines as Linkers in 2- and 3-Dimensional Architectures. CrystEngComm.

[11] Rocco D., Manfroni G., Prescimone A., Klein Y.M., Gawryluk D.J., Constable E.C., Housecroft C.E. (2020). Single and double-stranded 1D-coordination polymers with 4′-(4-alkyloxyphenyl)-3,2′:6′,3″-terpyridines and {Cu_2_(μ-OAc)_4_} or {Cu_4_(μ_3_-OH)_2_(μ-OAc)_2_(μ_3_-OAc)_2_(AcO-κ*O*)_2_} motifs. Polymers.

[12] Housecroft C.E. (2014). 4,2′:6′,4″-Terpyridines: Diverging and diverse building blocks in coordination polymers and metallomacrocycles. Dalton Trans..

[13] Housecroft C.E., Constable E.C. (2019). Ditopic and tetratopic 4,2′:6′,4″-Terpyridines as structural motifs in 2D- and 3D-coordination assemblies. Chimia.

[14] Yaghi O.M., O’Keeffe M., Ockwig N.W., Chae H.K., Eddaoudi M., Kim J. (2003). Reticular synthesis and the design of new materials. Nature.

[15] Liu Y., O’Keeffe M. (2018). Regular Figures, Minimal Transitivity, and Reticular Chemistry. Isr. J. Chem..

[16] Klein Y.M., Constable E.C., Housecroft C.E., Zampese J.A., Crochet A. (2014). Greasy tails switch 1D-coordination [Zn_2_(OAc)_4_(4′-(4-ROC_6_H_4_)-4,2′:6′,4″-tpy)]*_n_*polymers to discrete [Zn_2_(OAc)_4_(4′-(4-ROC_6_H_4_)-4,2′:6′,4″-tpy)_2_] complexes. Cryst. Eng. Comm..

[17] Constable E.C., Housecroft C.E., Vujovic S., Zampese J.A. (2014). 2D → 2D Parallel interpenetration of (4,4) sheets constructed from a ditopic bis(4,2′:6′,4″-terpyridine). Cryst. Eng. Comm..

[18] Vujovic S., Constable E.C., Housecroft C.E., Morris C.D., Neuburger M., Prescimone A. (2015). Engineering 2D→2D parallel interpenetration using long alkoxy-chain substituents. Polyhedron.

[19] Rocco D., Prescimone A., Klein Y.M., Gawryluk D.J., Constable E.C., Housecroft C.E. (2019). Competition in coordination assemblies: 1D-coordination polymer or 2D-nets based on Co(NCS)_2_ and 4′-(4-methoxyphenyl)-3,2′:6′,3″-terpyridine. Polymers.

[20] Rocco D., Housecroft C.E., Constable E.C. (2019). Synthesis of terpyridines: Simple reactions*—*What could possibly go wrong?. Molecules.

[21] Li L., Zhang Y.Z., Yang C., Liu E., Golen J.A., Zhang G. (2016). One-dimensional copper(II) coordination polymers built on 4′-substituted 4,2′:6′,4″- and 3,2′:6′,3″-terpyridines: Syntheses, structures and catalytic properties. Polyhedron.

[22] Cave G.W.V., Raston C.L. (2001). Efficient synthesis of pyridines via a sequential solventless aldol condensation and Michael addition. J. Chem. Soc. Perkin Trans. 1.

[23] Klein Y.M., Prescimone A., Constable E.C., Housecroft C.E. (2016). 2-Dimensional networks assembled using 4′-functionalized 4,2′:6′,4″-terpyridines and Co(NCS)_2_. Polyhedron.

[24] Klein Y.M., Prescimone A., Pitak M.B., Coles S.J., Constable E.C., Housecroft C.E. (2016). Constructing chiral MOFs by functionalizing 4,2′:6′,4″-terpyridine with long-chain alkoxy domains: Rare examples of *neb* nets. Cryst. Eng. Comm..

[25] SAINT (2013). Software for the Integration of CCD Detector System Bruker Analytical X-ray System.

[26] Sheldrick G.M. (2015). ShelXT-Integrated space-group and crystal-structure determination. Acta Cryst..

[27] Dolomanov O.V., Bourhis L.J., Gildea R.J., Howard J.A.K., Puschmann H. (2009). Olex2: A complete structure solution, refinement and analysis program. J. Appl. Cryst..

[28] Sheldrick G.M. (2015). Crystal structure refinement with ShelXL. Acta Cryst..

[29] Palatinus L., Chapuis G. (2007). Superflip*—*A computer program for the solution of crystal structures by charge flipping in arbitrary dimensions. J. Appl. Cryst..

[30] Palatinus L., Prathapa S.J., van Smaalen S. (2012). EDMA: A computer program for topological analysis of discrete electron densities. J. Appl. Cryst..

[31] Macrae C.F., Edgington P.R., McCabe P., Pidcock E., Shields G.P., Taylor R., Towler M., van de Streek J. (2006). Mercury: Visualization and analysis of crystal structures. J. Appl. Cryst..

[32] Macrae C.F., Bruno I.J., Chisholm J.A., Edgington P.R., McCabe P., Pidcock E., Rodriguez-Monge L., Taylor R., van de Streek J., Wood P.A. (2008). Mercury CSD 2.0*—*New Features for the visualization and investigation of crystal structures. J. Appl. Cryst..

[33] LeBail A., Duroy H., Fourquet J.L. (1988). Ab-initio structure determination of LiSbWO6 by X-ray powder diffraction. Mat. Res. Bull..

[34] Pawley G.S. (1981). Unit-cell refinement from powder diffraction scans. J. Appl. Cryst..

[35] Rodríguez-Carvajal J. (1993). Recent advances in magnetic structure determination by neutron powder diffraction. Phys. B.

[36] Roisnel T., Rodríguez-Carvajal J. WinPLOTR: A Windows tool for powder diffraction patterns analysis. Proceedings of the Seventh European Powder Diffraction Conference.

