# Supplementary material for: Directing 2D-Coordination Networks: Combined Effects of a Conformationally Flexible 3,2′:6′,3″-Terpyridine and Chain Length Variation in 4′-(4-n-Alkyloxyphenyl) Substituents
Source: Molecules. 2020 Apr 4;25(7):1663. doi: 10.3390/molecules25071663 (PMC7180911; doi:10.3390/molecules25071663)
Supplement: Supplementary file 1 [file molecules-25-01663-s001.zip › Supporting Information.pdf]

Supporting Information

Directing 2D-coordination networks: combined effects of a conformationally flexible 3,2':6',3''-terpyridine and chain length variation in 4'-(4-*n*-alkyloxyphenyl) substituents

Dalila Rocco [1], Alessandro Prescimone [1], Edwin C. Constable [1] and Catherine E. Housecroft [1,*]

[1] Department of Chemistry, University of Basel, BPR 1096, Mattenstrasse 24a, CH-4058 Basel, Switzerland; dalila.rocco@unibas.ch (D.R.); alessandro.prescimone@unibas.ch (A.P.); edwin.constable@unibas.ch (E.C.C.)

* Correspondence: catherine.housecroft@unibas.ch (C.E.H.); Tel: +41 61 207 1008

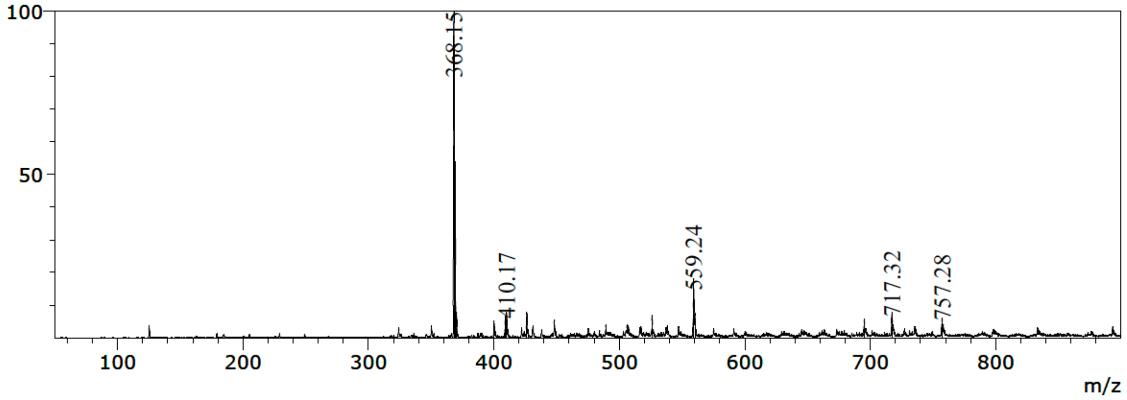

(a)

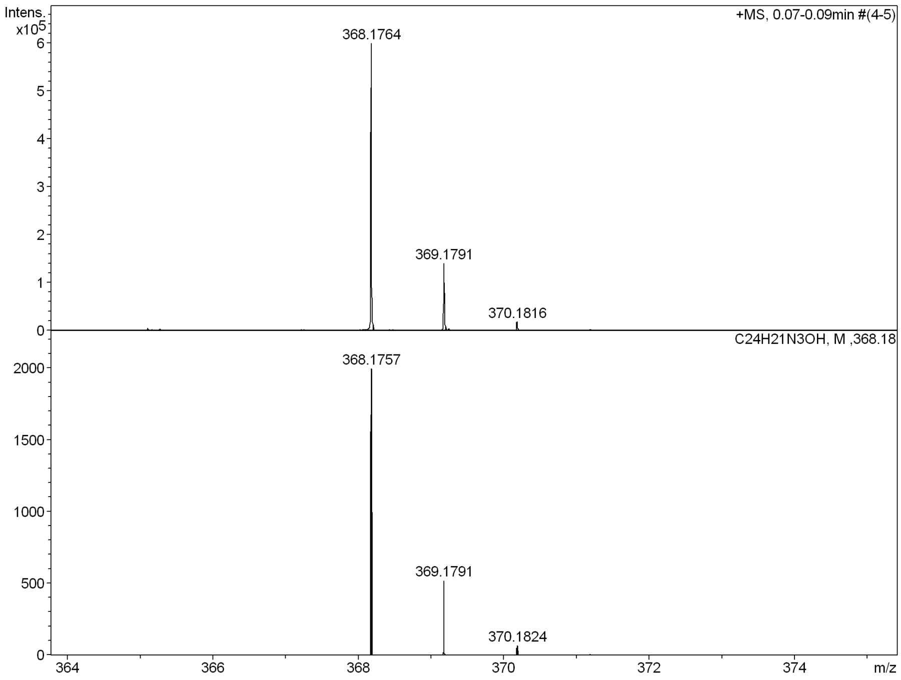

(b)

Figure S1. (a) Electrospray (ESI) mass spectrum of compound **3** showing the $[M+H]^+$ ion. (b) High-resolution (ESI) mass spectrum of **3** (top) with simulated spectrum (below).

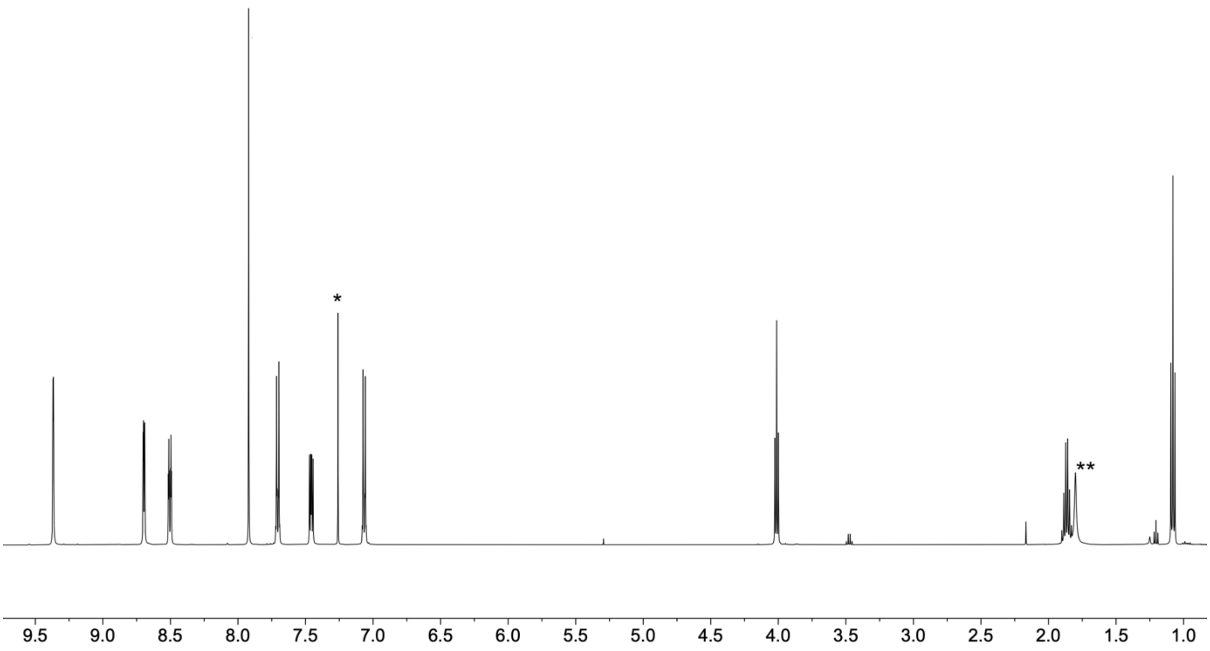

Figure S2. ^1H NMR (500 HMz, $CDCl_3$, 298 K) spectrum of **3**. * = residual $CHCl_3$, ** = H_2O.

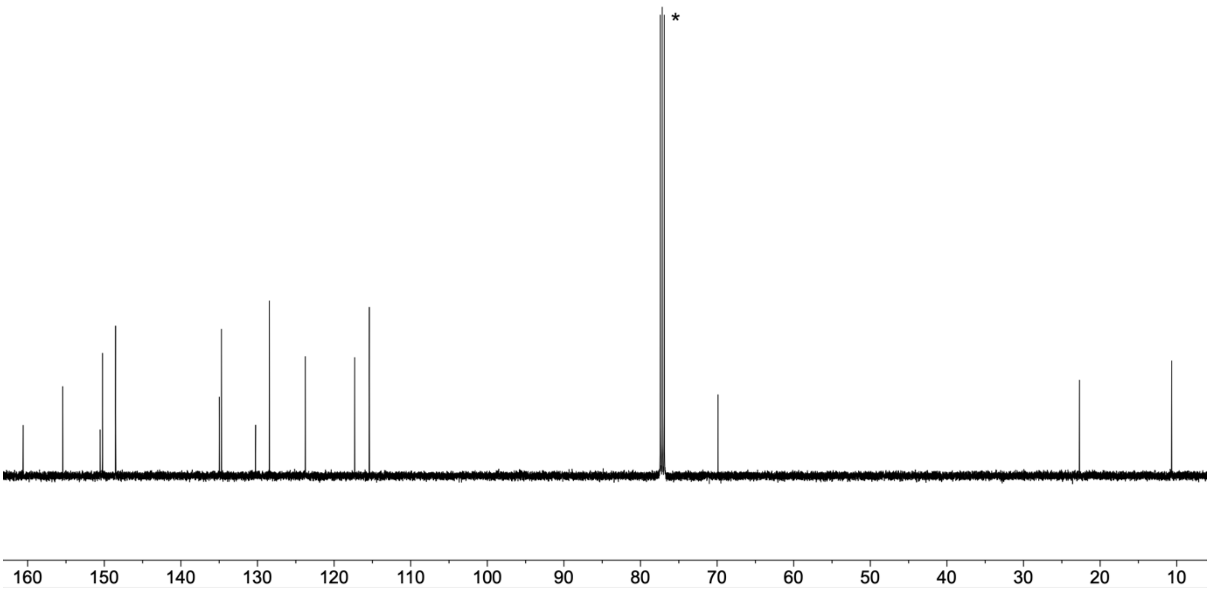

Figure S3. $^{13}C\{^1H\}$ NMR (126 HMz, $CDCl_3$, 298 K) spectrum of **3**. * = $CDCl_3$.

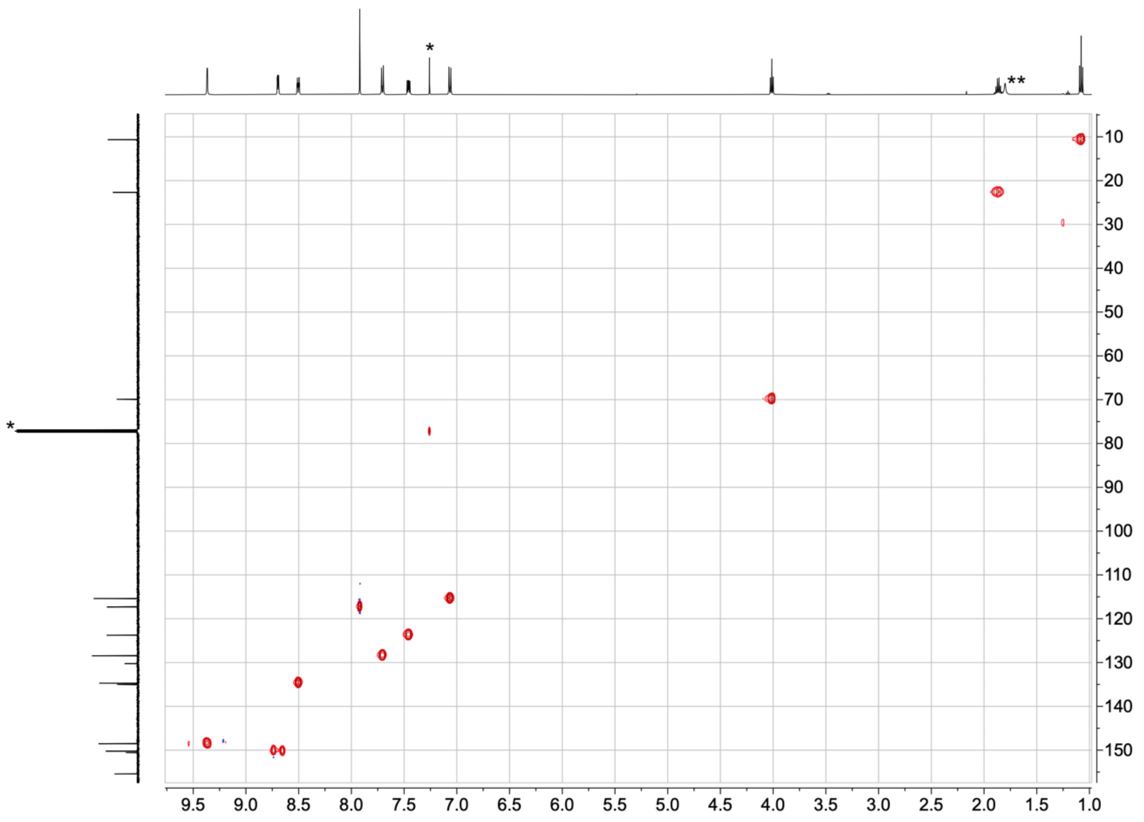

Figure S4. HMQC (^{1}H 500 MHz, ^{3}C{^{1}H} 126 HMz, $CDCl_3$, 298 K) spectrum of **3**. * = $CDCl_3$ or residual $CHCl_3$, ** = H_2O.

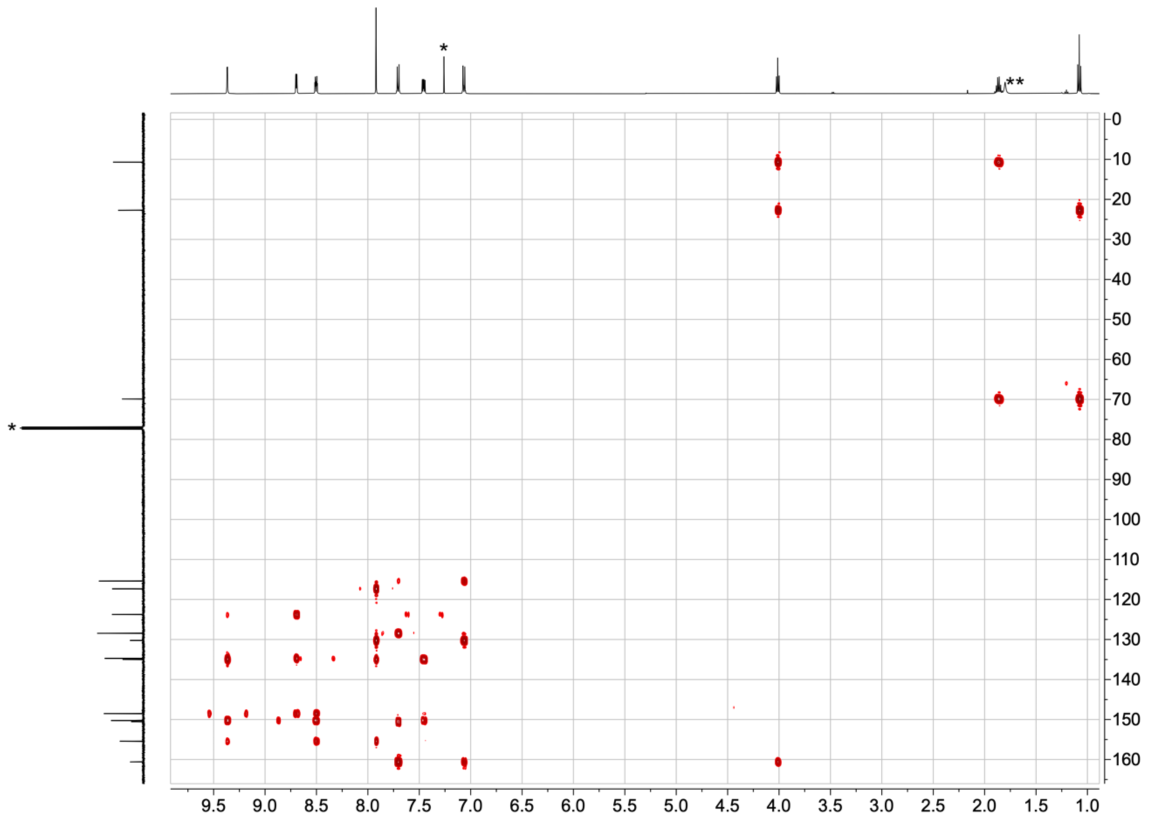

Figure S5. HMBC (^{1}H 500 MHz, ^{3}C{^{1}H} 126 HMz, $CDCl_3$, 298 K) spectrum of **3**. * = $CDCl_3$ or residual $CHCl_3$, ** = H_2O.

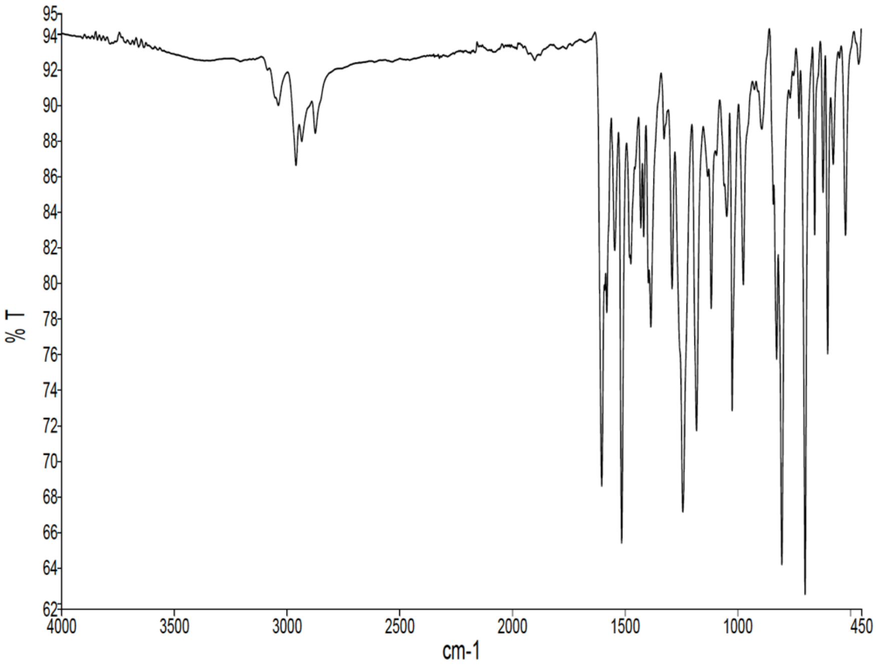

Figure S6. Solid-state IR spectrum of compound **3**.

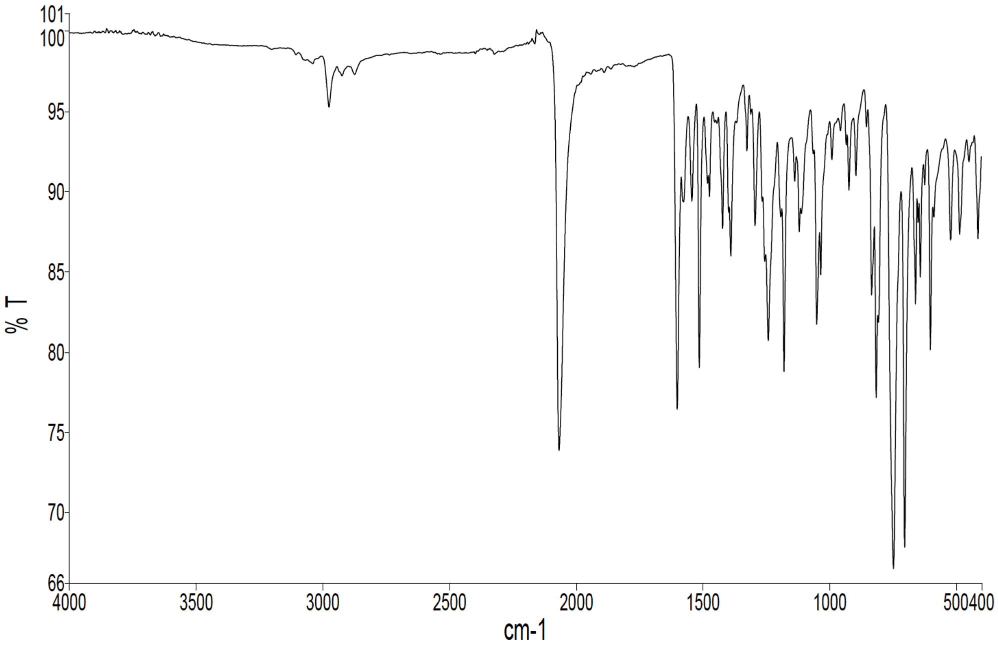

Figure S7. Solid-state IR spectrum of single crystals of $[\{Co(\mathbf{2})_2(NCS)_2\} \cdot 0.6CHCl_3]_n$.

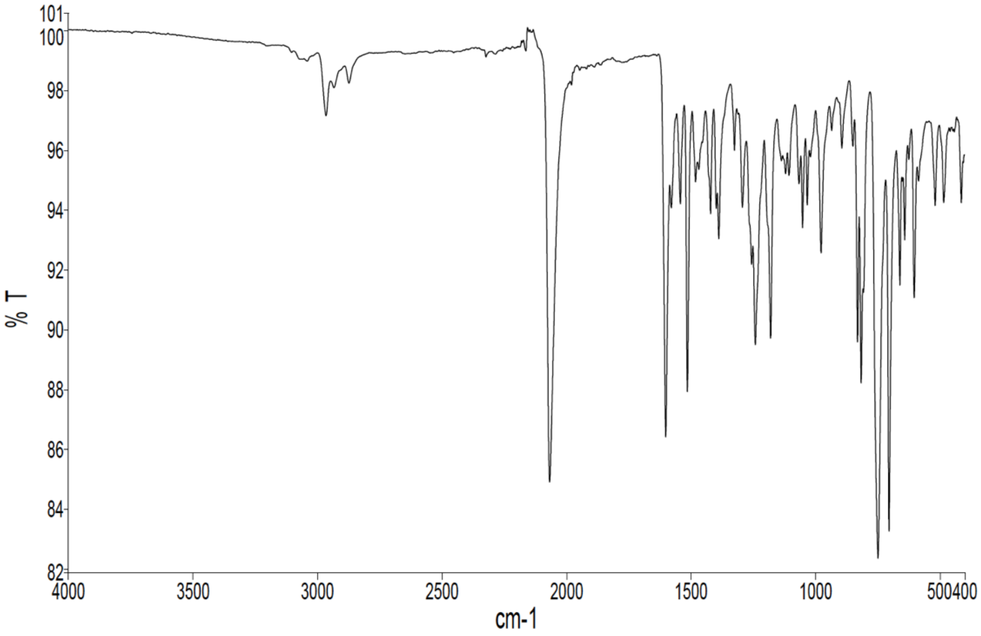

Figure S8. Solid-state IR spectrum of single crystals of [{Co(**3**)$_2$(NCS)$_2$}·4CHCl$_3$·0.25H$_2$O]$_n$.

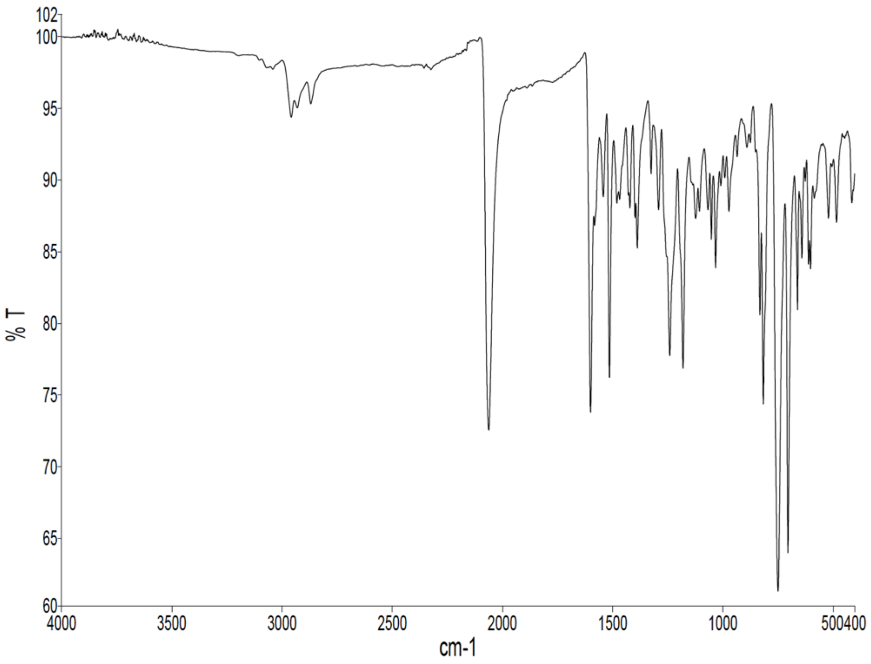

Figure S9. Solid-state IR spectrum of single crystals of [{Co(**4**)$_2$(NCS)$_2$}·4CHCl$_3$]$_n$.

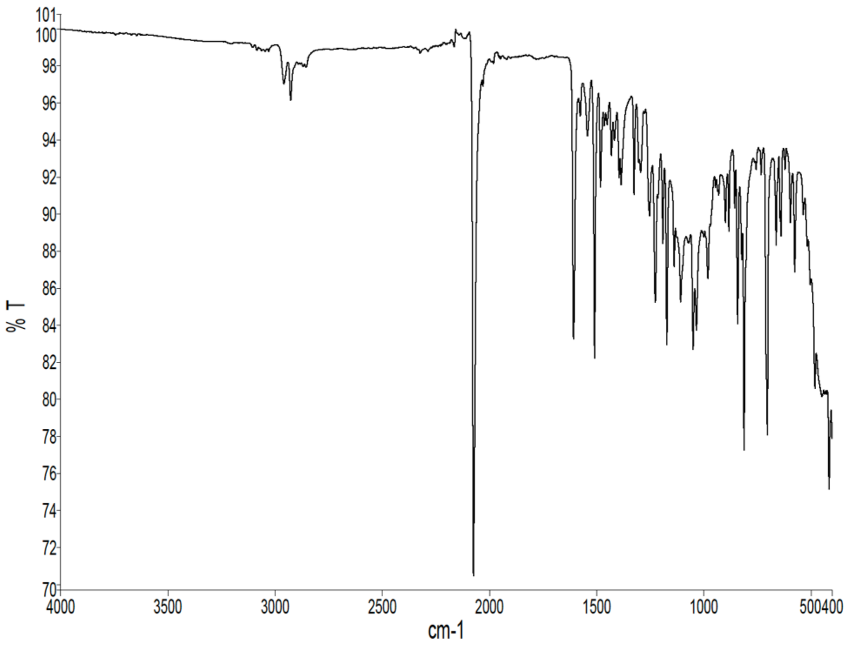

Figure S10. Solid-state IR spectrum of single crystals of $[Co_2(\mathbf{5})_4(NCS)_4]_n$.

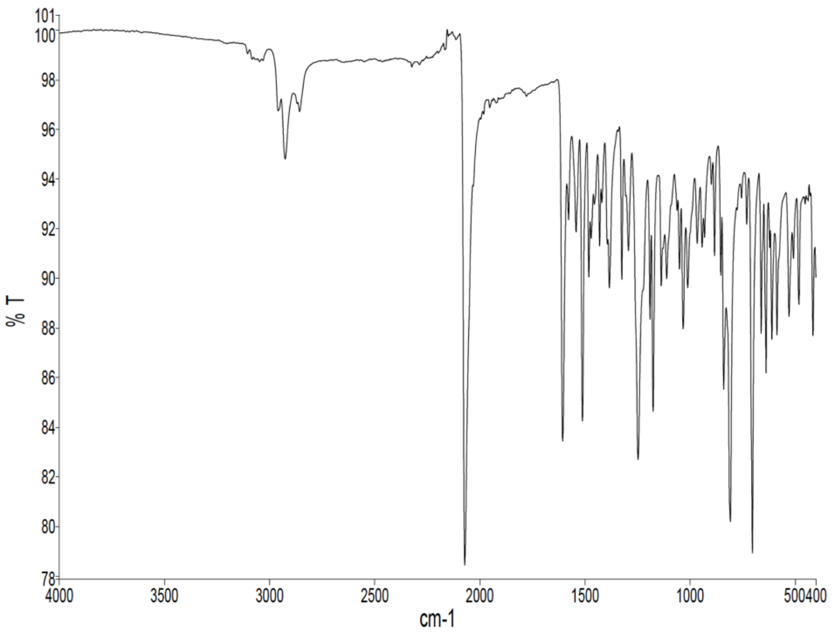

Figure S11. Solid-state IR spectrum of single crystals of $[Co(\mathbf{6})_2(NCS)_2]_n$.

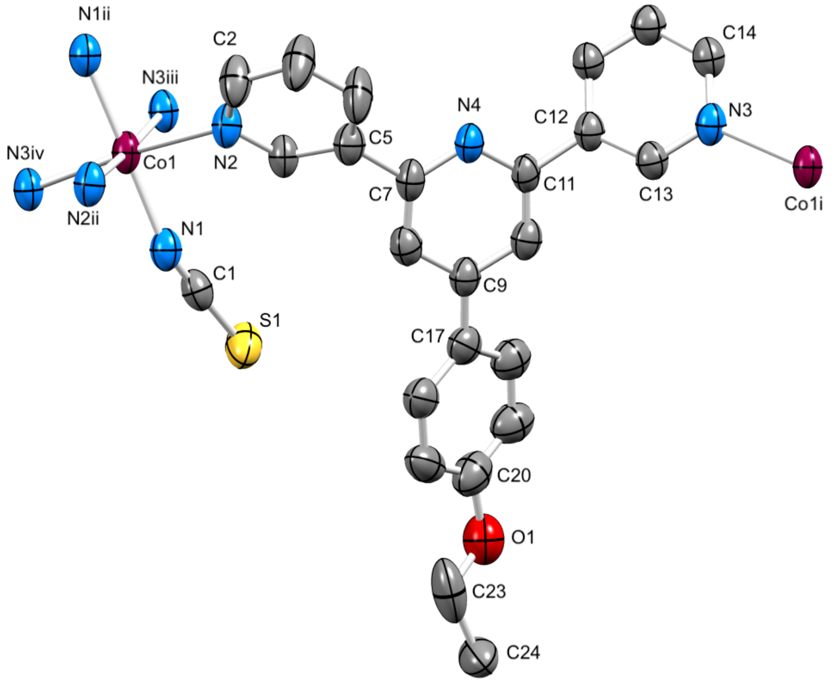

Figure S12. ORTEP representation of the asymmetric unit in [{Co(**2**)$_2$(NCS)$_2$}·0.6CHCl$_3$]$_n$ with symmetry generated atoms. Ellipsoids are plotted at a 40% probability level, and H atoms are omitted. Symmetry codes: i = $^1/_2$–*y*, *x*, *z*; ii = 1–*y*, 1–*x*, $^3/_2$–*z*; iii = *y*, $^1/_2$–*x*, *z*; iv = $^1/_2$+*x*, 1–*y*, $^3/_2$–*z*.

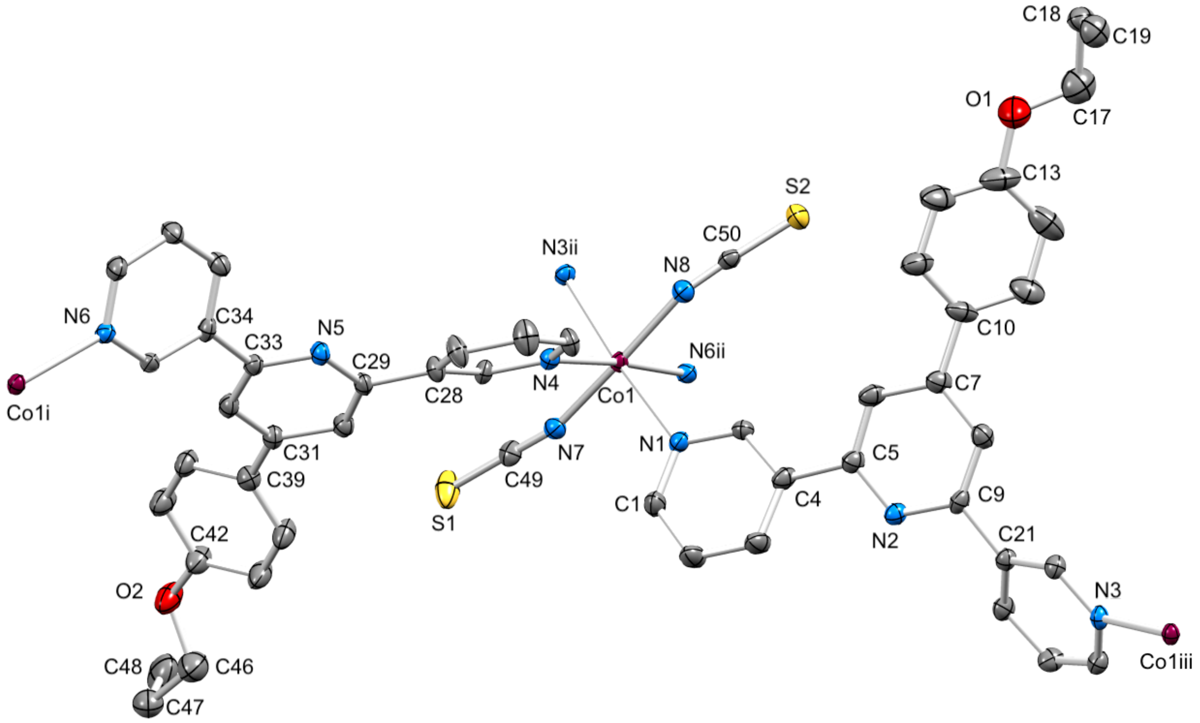

Figure S13. ORTEP representation of the asymmetric unit in [{Co(**3**)$_2$(NCS)$_2$}·4CHCl$_3$·0.25H$_2$O]$_n$ with symmetry generated atoms. Ellipsoids are plotted at a 40% probability level, and H atoms are omitted. Symmetry codes: i = $^1/_2$–*x*, –$^1/_2$+*y*, $^1/_2$–*z*; ii = $^1/_2$–*x*, $^1/_2$+*y*, $^1/_2$–*z*; iii = $^1/_2$+*x*, $^3/_2$–*y*, $^1/_2$–*z*.

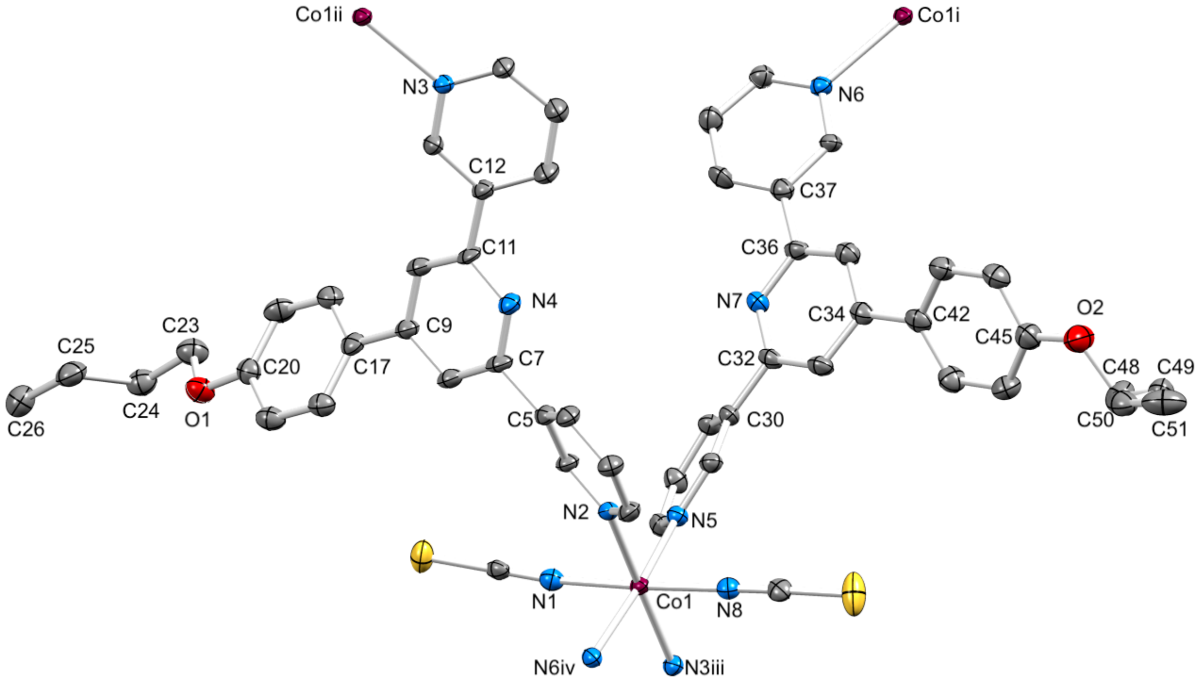

Figure S14. ORTEP representation of the asymmetric unit in $[\{Co(\mathbf{4})_2(NCS)_2\}{\cdot}4CHCl_3]_n$ with symmetry generated atoms. Ellipsoids are plotted at a 40% probability level, and H atoms omitted. Symmetry codes: i = $^{3}/_{2}-x$, $^{1}/_{2}+y$, $^{1}/_{2}-z$; ii = $-^{1}/_{2}+x$, $^{1}/_{2}-y$, $^{1}/_{2}-z$; iii = $^{1}/_{2}+x$, $^{1}/_{2}-y$, $^{1}/_{2}-z$; iv = $^{3}/_{2}-x$, $-^{1}/_{2}+y$, $^{1}/_{2}-z$.

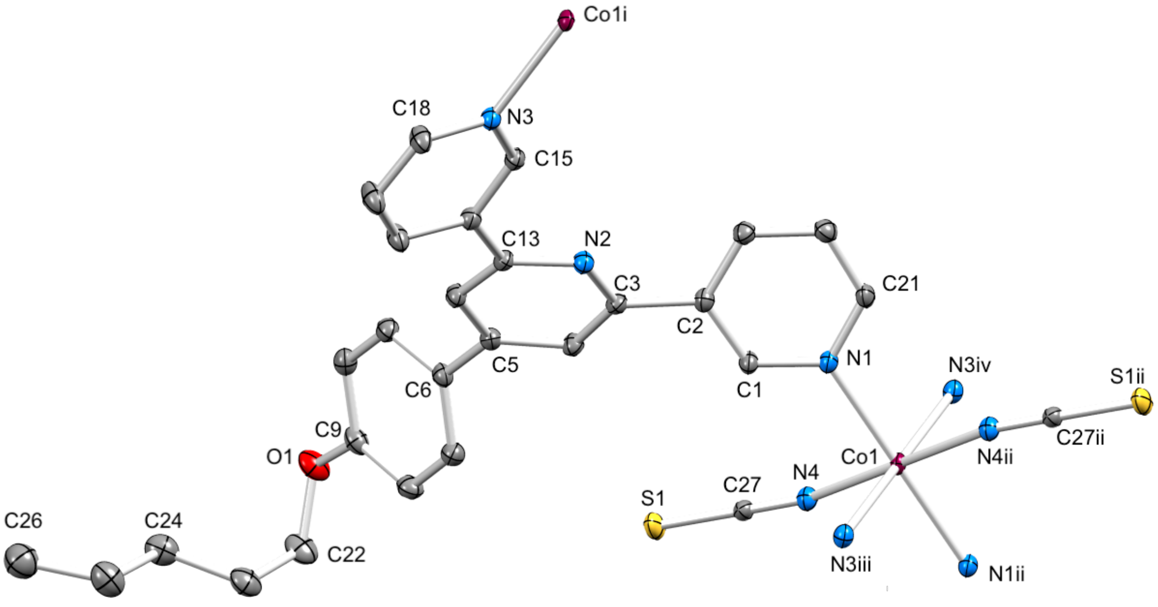

Figure S15. ORTEP representation of the asymmetric unit in $[Co_2(\mathbf{5})_4(NCS)_4]_n$ with symmetry generated atoms. Ellipsoids are plotted at a 40% probability level, and H atoms omitted. Symmetry codes: i = $^{1}/_{2}-x$, $-^{1}/_{2}+y$, $^{3}/_{2}-z$; ii = $1-x$, $1-y$, $2-z$; iii = $^{1}/_{2}+x$, $^{1}/_{2}-y$, $^{1}/_{2}+z$; iv = $^{1}/_{2}-x$, $^{1}/_{2}+y$, $^{3}/_{2}+z$.

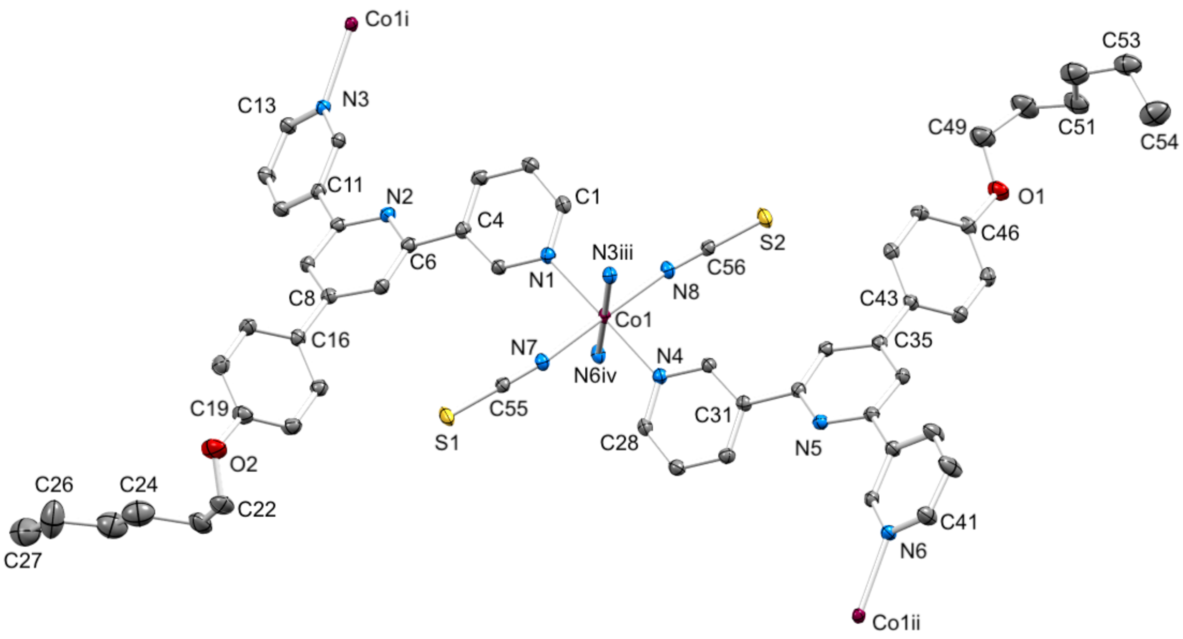

Figure S16. ORTEP representation of the asymmetric unit in [Co(**6**)$_2$(NCS)$_2$]$_n$ with symmetry generated atoms. Ellipsoids are plotted at a 40% probability level, and H atoms omitted. Symmetry codes: i = 2–*x*, –$^1/_2$+*y*, $^3/_2$–*z*; ii = 1–*x*, $^1/_2$+*y*, $^3/_2$–*z*; iii = 2–*x*, $^1/_2$+*y*, $^3/_2$–*z*; iv = 1–*x*, –$^1/_2$+*y*, $^3/_2$–*z*.

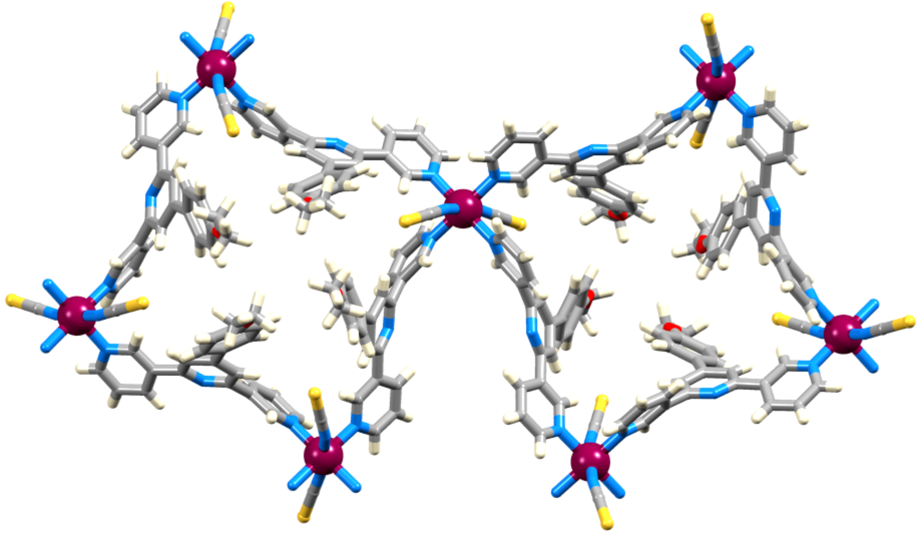

Figure S17. Detail of the relative orientations of 4-ethoxyphenyl around the edges of two corner-sharing squares in the (4,4) net in [{Co(**2**)$_2$(NCS)$_2$}·0.6CHCl$_3$]$_n$.

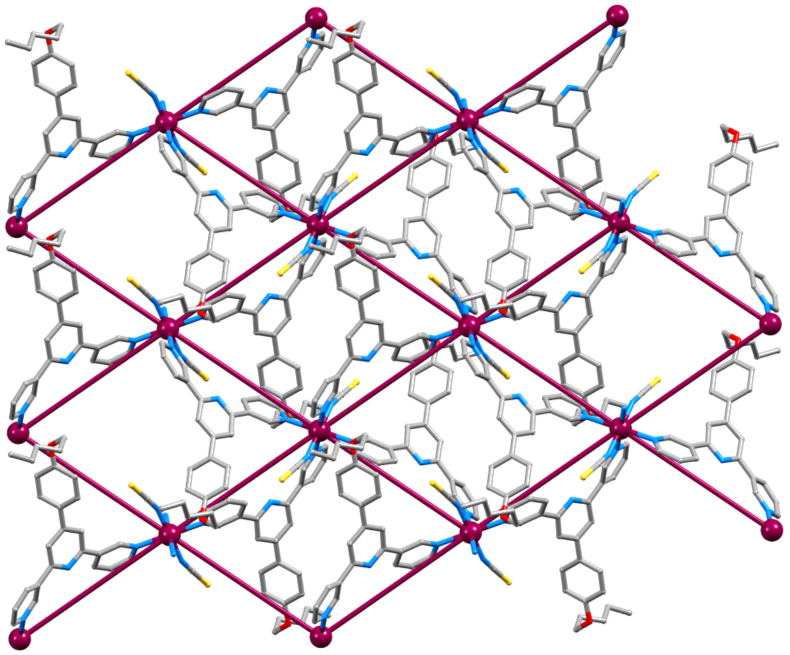

Figure S18. Part of one (4,4) net in $[Co_2(\mathbf{5})_4(NCS)_4\}]_n$ with the net defined by the lines connecting the Co atoms; H atoms are omitted.

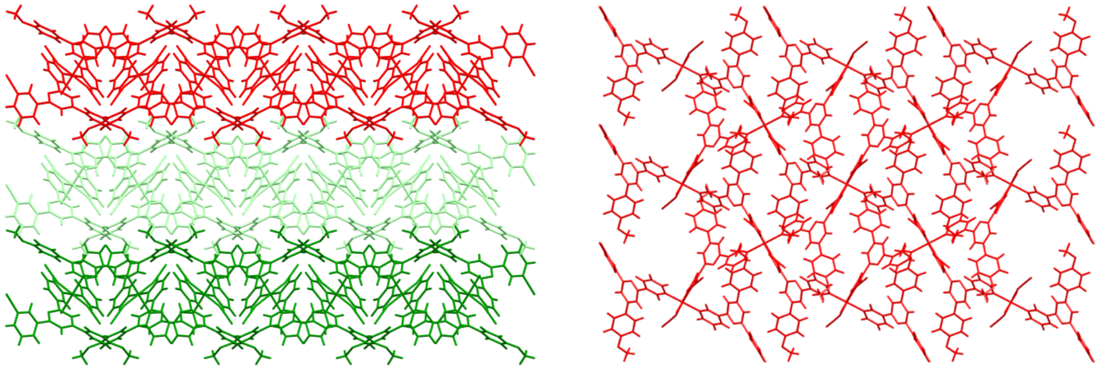

(a) (b)

Figure S19. Eclipsed 2D-sheets in $[\{Co(\mathbf{1})_2(NCS)_2\}\cdot 3MeOH]_n$: (a) view down the *c*-axis, and (b) view down the *a*-axis. CSD refcode FOXQUH.
