# Supplementary material for: Directing 2D-Coordination Networks: Combined Effects of a Conformationally Flexible 3,2′:6′,3″-Terpyridine and Chain Length Variation in 4′-(4-n-Alkyloxyphenyl) Substituents
Source: Molecules. 2020 Apr 4;25(7):1663. doi: 10.3390/molecules25071663 (PMC7180911; doi:10.3390/molecules25071663)

checkCIF/PLATON report

Structure factors have been supplied for datablock(s) rod075a_130k_0m

THIS REPORT IS FOR GUIDANCE ONLY. IF USED AS PART OF A REVIEW PROCEDURE FOR PUBLICATION, IT SHOULD NOT REPLACE THE EXPERTISE OF AN EXPERIENCED CRYSTALLOGRAPHIC REFEREE.

No syntax errors found.   CIF dictionary   Interpreting this report

Datablock: rod075a_130k_0m

```
Bond precision:  C-C = 0.0122 A          Wavelength=1.54178

Cell:            a=25.6618(13)     b=25.6618(13)     c=18.709(1)
                 alpha=90          beta=90           gamma=90
Temperature:     130 K
```

	Calculated	Reported
Volume	12320.4(14)	12320.4(14)
Space group	P -4 21 c	P -4 21 c
Hall group	P -4 2n	P -4 2n
Moiety formula	4(C50 H42 Co N8 O2 S2), 16(C H Cl3), H2 O	C50 H42 Co N8 O2 S2, 4(C H Cl3), 0.25(H2 O)
Sum formula	C216 H186 Cl48 Co4 N32 O9 S8	C54 H46.50 Cl12 Co N8 O2.25 S2
Mr	5567.77	1391.94
Dx,g cm-3	1.501	1.501
Z	2	8
Mu (mm-1)	7.998	7.998
F000	5660.0	5660.0
F000'	5692.36	
h,k,lmax	31,31,22	30,30,22
Nref	11726[6334]	11345
Tmin,Tmax	0.202,0.449	0.522,0.753
Tmin'	0.127	

```
Correction method= # Reported T Limits: Tmin=0.522 Tmax=0.753
AbsCorr = MULTI-SCAN

Data completeness= 1.79/0.97      Theta(max)= 70.181

R(reflections)= 0.0684( 10789)    wR2(reflections)= 0.1998( 11345)

S = 1.115                  Npar= 720
```

The following ALERTS were generated. Each ALERT has the format
test-name_ALERT_alert-type_alert-level.
Click on the hyperlinks for more details of the test.

Alert level B

```
PLAT109_ALERT_2_B Twinning Matrix is inverted Laue group operation          ? Check
```

Alert level C

```
PLAT242_ALERT_2_C Low    MainMol Ueq as Compared to Neighbors of        C18 Check
PLAT244_ALERT_4_C Low    Solvent Ueq as Compared to Neighbors of        C51 Check
PLAT244_ALERT_4_C Low    Solvent Ueq as Compared to Neighbors of        C53 Check
PLAT341_ALERT_3_C Low Bond Precision on  C-C Bonds ...............    0.01215 Ang.
PLAT911_ALERT_3_C Missing FCF Refl Between Thmin & STh/L=    0.600         41 Report
PLAT918_ALERT_3_C Reflection(s) with I(obs) much Smaller I(calc) .          2 Check
```

Alert level G

```
PLAT003_ALERT_2_G Number of Uiso or Uij Restrained non-H Atoms ...          1 Report
PLAT004_ALERT_5_G Polymeric Structure Found with Maximum Dimension          2 Info
PLAT042_ALERT_1_G Calc. and Reported MoietyFormula Strings  Differ     Please Check
PLAT045_ALERT_1_G Calculated and Reported Z Differ by a Factor ...       0.25 Check
PLAT072_ALERT_2_G SHELXL First  Parameter in WGHT  Unusually Large       0.14 Report
PLAT083_ALERT_2_G SHELXL Second Parameter in WGHT  Unusually Large      13.30 Why ?
PLAT186_ALERT_4_G The CIF-Embedded .res File Contains ISOR Records          1 Report
PLAT300_ALERT_4_G Atom Site Occupancy of O0AA       Constrained at        0.5 Check
PLAT300_ALERT_4_G Atom Site Occupancy of H0AA       Constrained at       0.25 Check
PLAT300_ALERT_4_G Atom Site Occupancy of H0AB       Constrained at       0.25 Check
PLAT302_ALERT_4_G Anion/Solvent/Minor-Residue Disorder (Resd  6  )       100% Note
PLAT434_ALERT_2_G Short Inter HL..HL Contact Cl1      ..Cl12             3.35 Ang.
                                               x,y,-1+z  =      1_554 Check
PLAT720_ALERT_4_G Number of Unusual/Non-Standard Labels ..........          3 Note
PLAT794_ALERT_5_G Tentative Bond Valency for Co1       (II)      .       1.89 Info
PLAT860_ALERT_3_G Number of Least-Squares Restraints .............          6 Note
PLAT870_ALERT_4_G ALERTS Related to Twinning Effects Suppressed ..          ! Info
PLAT910_ALERT_3_G Missing # of FCF Reflection(s) Below Theta(Min).          1 Note
PLAT912_ALERT_4_G Missing # of FCF Reflections Above STh/L=  0.600         98 Note
PLAT916_ALERT_2_G Hooft y and Flack x Parameter Values Differ by .       0.46 Check
PLAT931_ALERT_5_G CIFcalcFCF  Twin Law ( 1 1 0)          Est.d BASF      0.45 Check
PLAT933_ALERT_2_G Number of OMIT Records in Embedded .res File ...          1 Note
PLAT992_ALERT_5_G Repd & Actual _reflns_number_gt Values Differ by          1 Check
```

---

0 **ALERT level A** = Most likely a serious problem - resolve or explain
1 **ALERT level B** = A potentially serious problem, consider carefully
6 **ALERT level C** = Check. Ensure it is not caused by an omission or oversight
22 **ALERT level G** = General information/check it is not something unexpected

2 ALERT type 1 CIF construction/syntax error, inconsistent or missing data
8 ALERT type 2 Indicator that the structure model may be wrong or deficient
5 ALERT type 3 Indicator that the structure quality may be low
10 ALERT type 4 Improvement, methodology, query or suggestion
4 ALERT type 5 Informative message, check

It is advisable to attempt to resolve as many as possible of the alerts in all categories. Often the minor alerts point to easily fixed oversights, errors and omissions in your CIF or refinement strategy, so attention to these fine details can be worthwhile. In order to resolve some of the more serious problems it may be necessary to carry out additional measurements or structure refinements. However, the purpose of your study may justify the reported deviations and the more serious of these should normally be commented upon in the discussion or experimental section of a paper or in the "special_details" fields of the CIF. checkCIF was carefully designed to identify outliers and unusual parameters, but every test has its limitations and alerts that are not important in a particular case may appear. Conversely, the absence of alerts does not guarantee there are no aspects of the results needing attention. It is up to the individual to critically assess their own results and, if necessary, seek expert advice.

Publication of your CIF in IUCr journals

A basic structural check has been run on your CIF. These basic checks will be run on all CIFs submitted for publication in IUCr journals (*Acta Crystallographica*, *Journal of Applied Crystallography*, *Journal of Synchrotron Radiation*); however, if you intend to submit to *Acta Crystallographica Section C* or *E* or *IUCrData*, you should make sure that full publication checks are run on the final version of your CIF prior to submission.

Publication of your CIF in other journals

Please refer to the *Notes for Authors* of the relevant journal for any special instructions relating to CIF submission.

PLATON version of 22/12/2019; check.def file version of 13/12/2019

Datablock rod075a_130k_0m - ellipsoid plot

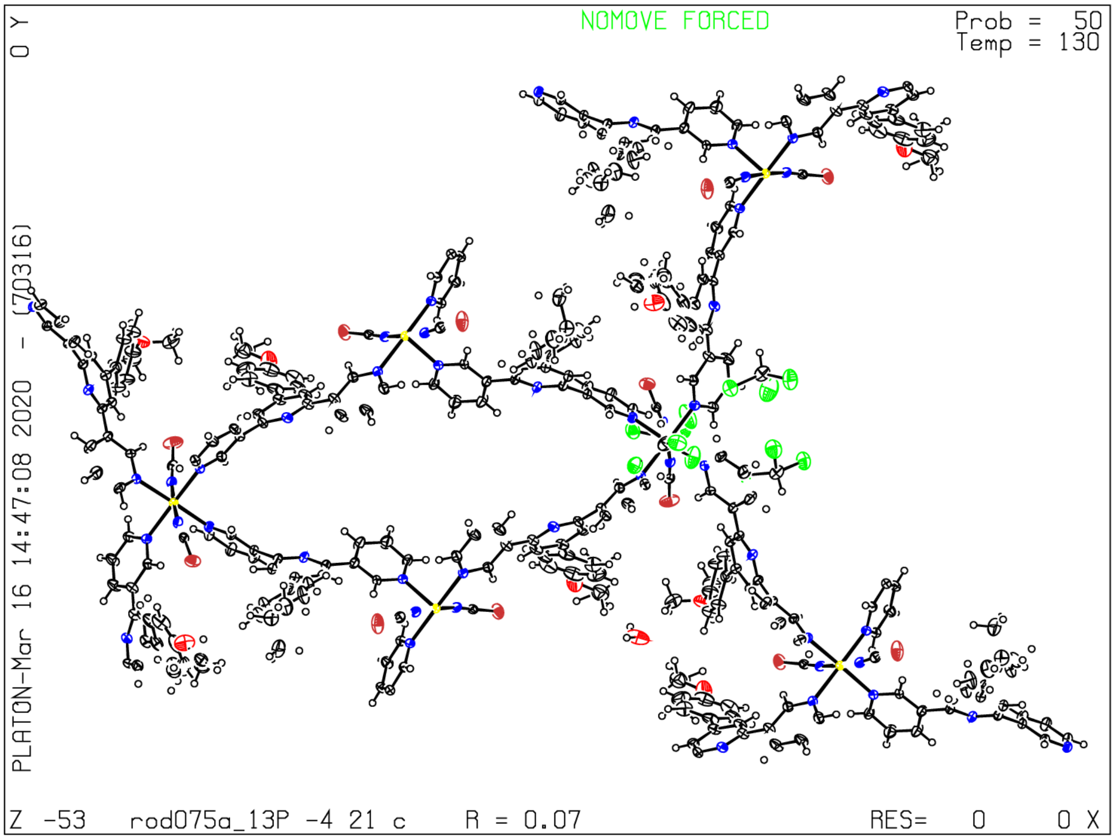

Supplement: Supplementary file 1 [file molecules-25-01663-s001.zip › ROD075A_130K_0m_cifreport.pdf]
