# Supplementary material for: Directing 2D-Coordination Networks: Combined Effects of a Conformationally Flexible 3,2′:6′,3″-Terpyridine and Chain Length Variation in 4′-(4-n-Alkyloxyphenyl) Substituents
Source: Molecules. 2020 Apr 4;25(7):1663. doi: 10.3390/molecules25071663 (PMC7180911; doi:10.3390/molecules25071663)

checkCIF/PLATON report

Structure factors have been supplied for datablock(s) rod059b_130k

THIS REPORT IS FOR GUIDANCE ONLY. IF USED AS PART OF A REVIEW PROCEDURE FOR PUBLICATION, IT SHOULD NOT REPLACE THE EXPERTISE OF AN EXPERIENCED CRYSTALLOGRAPHIC REFEREE.

No syntax errors found. CIF dictionary Interpreting this report

Datablock: rod059b_130k

```
Bond precision:  C-C = 0.0034 A          Wavelength=1.34143

Cell:            a=13.1357(4)    b=12.4666(3)        c=15.1441(4)
                 alpha=90        beta=100.148(2)     gamma=90
Temperature:     130 K

                Calculated                Reported
Volume          2441.16(12)               2441.16(12)
Space group     P 21/n                    P 1 21/n 1
Hall group      -P 2yn                    -P 2yn
Moiety formula  C54 H50 Co N8 O2 S2       C108 H100 Co2 N16 O4 S4
Sum formula     C54 H50 Co N8 O2 S2       C108 H100 Co2 N16 O4 S4
Mr              966.07                    1932.13
Dx,g cm-3       1.314                     1.314
Z               2                         1
Mu (mm-1)       2.710                     2.675
F000            1010.0                    1010.0
F000'           1011.70
h,k,lmax        16,15,18                  16,15,18
Nref            5021                      4983
Tmin,Tmax       0.670,0.899               1.000,1.000
Tmin'           0.558

Correction method= # Reported T Limits: Tmin=1.000 Tmax=1.000
AbsCorr = MULTI-SCAN

Data completeness= 0.992          Theta(max)= 57.080

R(reflections)= 0.0500( 4596)     wR2(reflections)= 0.1453( 4983)

S = 1.113                  Npar= 305
```

The following ALERTS were generated. Each ALERT has the format
test-name_ALERT_alert-type_alert-level.
Click on the hyperlinks for more details of the test.

Alert level B

```
PLAT934_ALERT_3_B Number of (Iobs-Icalc)/Sigma(W) > 10 Outliers ..          2 Check
```

Alert level C

```
PLAT051_ALERT_1_C Mu(calc) and Mu(CIF) Ratio Differs from 1.0 by .       1.31 %
PLAT911_ALERT_3_C Missing FCF Refl Between Thmin & STh/L=    0.600          4 Report
PLAT918_ALERT_3_C Reflection(s) with I(obs) much Smaller I(calc) .          3 Check
PLAT978_ALERT_2_C Number C-C Bonds with Positive Residual Density.          0 Info
```

Alert level G

```
ABSMU01_ALERT_1_G  Calculation of _exptl_absorpt_correction_mu
                not performed for this radiation type.
PLAT004_ALERT_5_G Polymeric Structure Found with Maximum Dimension          2 Info
PLAT042_ALERT_1_G Calc. and Reported MoietyFormula Strings  Differ     Please Check
PLAT045_ALERT_1_G Calculated and Reported Z Differ by a Factor ...       2.00 Check
PLAT232_ALERT_2_G Hirshfeld Test Diff (M-X)  Co1      --N4       .        5.8 s.u.
PLAT794_ALERT_5_G Tentative Bond Valency for Co1       (II)      .       1.54 Info
PLAT910_ALERT_3_G Missing # of FCF Reflection(s) Below Theta(Min).          1 Note
PLAT912_ALERT_4_G Missing # of FCF Reflections Above STh/L=  0.600         33 Note
PLAT933_ALERT_2_G Number of OMIT Records in Embedded .res File ...          4 Note
PLAT984_ALERT_1_G The  C-f'=    0.0148 Deviates from the B&C-Value     0.0137 Check
PLAT984_ALERT_1_G The Co-f'=   -0.6673 Deviates from the B&C-Value    -0.6628 Check
PLAT984_ALERT_1_G The  N-f'=    0.0253 Deviates from the B&C-Value     0.0241 Check
PLAT984_ALERT_1_G The  O-f'=    0.0412 Deviates from the B&C-Value     0.0389 Check
PLAT985_ALERT_1_G The Co-f"=    2.8829 Deviates from the B&C-Value     2.9049 Check
PLAT985_ALERT_1_G The  S-f"=    0.4248 Deviates from the B&C-Value     0.4295 Check
PLAT992_ALERT_5_G Repd & Actual _reflns_number_gt Values Differ by          1 Check
```

```
   0 ALERT level A = Most likely a serious problem - resolve or explain
   1 ALERT level B = A potentially serious problem, consider carefully
   4 ALERT level C = Check. Ensure it is not caused by an omission or oversight
  16 ALERT level G = General information/check it is not something unexpected

  10 ALERT type 1 CIF construction/syntax error, inconsistent or missing data
   3 ALERT type 2 Indicator that the structure model may be wrong or deficient
   4 ALERT type 3 Indicator that the structure quality may be low
   1 ALERT type 4 Improvement, methodology, query or suggestion
   3 ALERT type 5 Informative message, check
```

Validation response form

Please find below a validation response form (VRF) that can be filled in and pasted into your CIF.

```
# start Validation Reply Form
_vrf_PLAT051_rod059b_130k
;
PROBLEM: Mu(calc) and Mu(CIF) Ratio Differs from 1.0 by .       1.31 %
RESPONSE: ...
;
_vrf_PLAT911_rod059b_130k
;
PROBLEM: Missing FCF Refl Between Thmin & STh/L=    0.600          4 Report
RESPONSE: ...
;
_vrf_PLAT918_rod059b_130k
;
PROBLEM: Reflection(s) with I(obs) much Smaller I(calc) .          3 Check
```

```
RESPONSE: ...
;
_vrf_PLAT978_rod059b_130k
;
PROBLEM: Number C-C Bonds with Positive Residual Density.          0 Info
RESPONSE: ...
;
# end Validation Reply Form
```

It is advisable to attempt to resolve as many as possible of the alerts in all categories. Often the minor alerts point to easily fixed oversights, errors and omissions in your CIF or refinement strategy, so attention to these fine details can be worthwhile. In order to resolve some of the more serious problems it may be necessary to carry out additional measurements or structure refinements. However, the purpose of your study may justify the reported deviations and the more serious of these should normally be commented upon in the discussion or experimental section of a paper or in the "special_details" fields of the CIF. checkCIF was carefully designed to identify outliers and unusual parameters, but every test has its limitations and alerts that are not important in a particular case may appear. Conversely, the absence of alerts does not guarantee there are no aspects of the results needing attention. It is up to the individual to critically assess their own results and, if necessary, seek expert advice.

Publication of your CIF in IUCr journals

A basic structural check has been run on your CIF. These basic checks will be run on all CIFs submitted for publication in IUCr journals (*Acta Crystallographica*, *Journal of Applied Crystallography*, *Journal of Synchrotron Radiation*); however, if you intend to submit to *Acta Crystallographica Section C* or *E* or *IUCrData*, you should make sure that full publication checks are run on the final version of your CIF prior to submission.

Publication of your CIF in other journals

Please refer to the *Notes for Authors* of the relevant journal for any special instructions relating to CIF submission.

PLATON version of 07/08/2019; check.def file version of 30/07/2019

Datablock rod059b_130k - ellipsoid plot

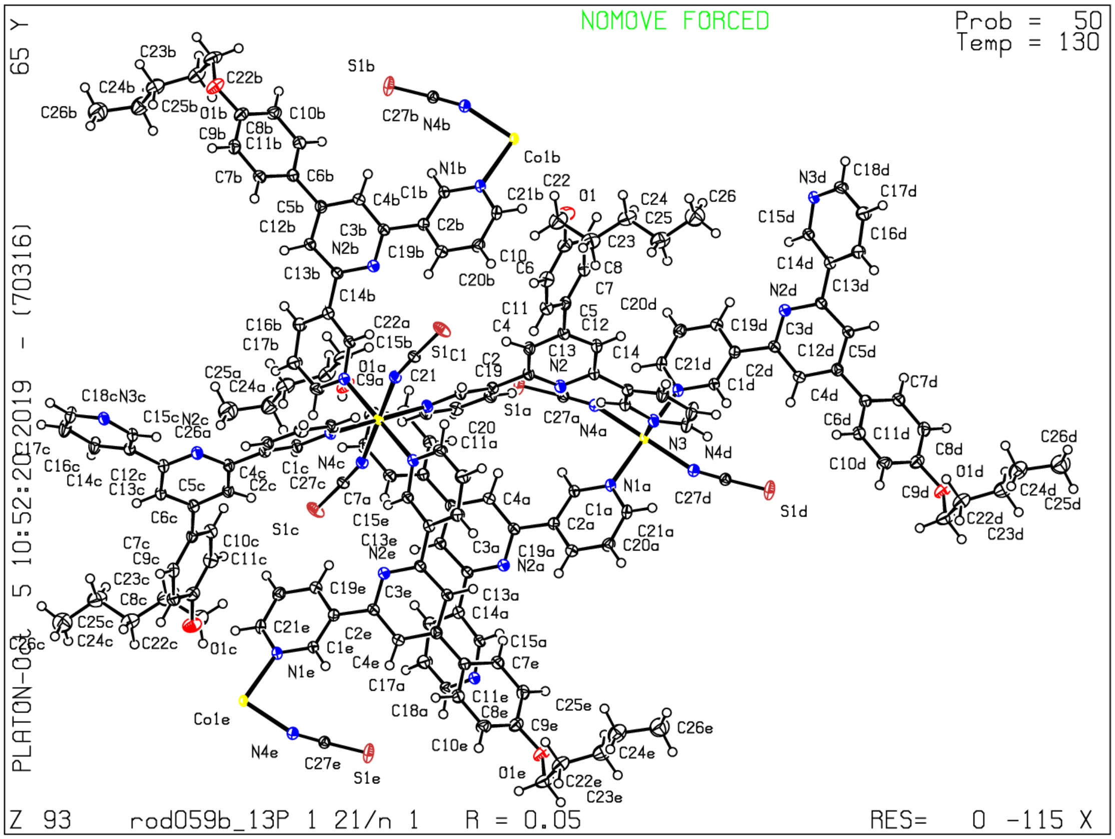

Supplement: Supplementary file 1 [file molecules-25-01663-s001.zip › ROD059B_checkcif.pdf]
