# Supplementary material for: Directing 2D-Coordination Networks: Combined Effects of a Conformationally Flexible 3,2′:6′,3″-Terpyridine and Chain Length Variation in 4′-(4-n-Alkyloxyphenyl) Substituents
Source: Molecules. 2020 Apr 4;25(7):1663. doi: 10.3390/molecules25071663 (PMC7180911; doi:10.3390/molecules25071663)

checkCIF/PLATON report

Structure factors have been supplied for datablock(s) p21c

THIS REPORT IS FOR GUIDANCE ONLY. IF USED AS PART OF A REVIEW PROCEDURE FOR PUBLICATION, IT SHOULD NOT REPLACE THE EXPERTISE OF AN EXPERIENCED CRYSTALLOGRAPHIC REFEREE.

No syntax errors found. CIF dictionary Interpreting this report

Datablock: p21c

```
Bond precision:  C-C = 0.0035 A          Wavelength=1.54178

Cell:           a=18.3988(14)    b=12.9419(10)     c=21.7046(17)
                alpha=90         beta=98.421(4)    gamma=90
Temperature:    130 K

                Calculated                 Reported
Volume          5112.5(7)                  5112.5(7)
Space group     P 21/c                     P 1 21/c 1
Hall group      -P 2ybc                    -P 2ybc
Moiety formula  C56 H54 Co N8 O2 S2        C56 H54 Co N8 O2 S2
Sum formula     C56 H54 Co N8 O2 S2        C56 H54 Co N8 O2 S2
Mr              994.12                     994.12
Dx,g cm-3       1.292                      1.292
Z               4                          4
Mu (mm-1)       3.787                      3.787
F000            2084.0                     2084.0
F000'           2082.35
h,k,lmax        22,15,26                   21,15,26
Nref            9752                       9388
Tmin,Tmax       0.500,0.767                0.623,0.753
Tmin'           0.378

Correction method= # Reported T Limits: Tmin=0.623 Tmax=0.753
AbsCorr = MULTI-SCAN

Data completeness= 0.963          Theta(max)= 70.232

R(reflections)= 0.0467( 8264)     wR2(reflections)= 0.1298( 9388)

S = 1.047                   Npar= 624
```

The following ALERTS were generated. Each ALERT has the format
test-name_ALERT_alert-type_alert-level.
Click on the hyperlinks for more details of the test.

Alert level C

```
PLAT094_ALERT_2_C Ratio of Maximum / Minimum Residual Density ....       3.22 Report
PLAT220_ALERT_2_C Non-Solvent  Resd 1  C   Ueq(max)/Ueq(min) Range        4.2 Ratio
PLAT222_ALERT_3_C Non-Solv.  Resd 1  H   Uiso(max)/Uiso(min) Range        4.4 Ratio
PLAT911_ALERT_3_C Missing FCF Refl Between Thmin & STh/L=    0.600         75 Report
PLAT971_ALERT_2_C Check Calcd Resid. Dens.  1.03A   From C24             1.55 eA-3
```

Alert level G

```
PLAT004_ALERT_5_G Polymeric Structure Found with Maximum Dimension          2 Info
PLAT720_ALERT_4_G Number of Unusual/Non-Standard Labels ..........          1 Note
PLAT794_ALERT_5_G Tentative Bond Valency for Co01      (II)      .       1.52 Info
PLAT912_ALERT_4_G Missing # of FCF Reflections Above STh/L=  0.600        264 Note
PLAT933_ALERT_2_G Number of OMIT Records in Embedded .res File ...          4 Note
PLAT978_ALERT_2_G Number C-C Bonds with Positive Residual Density.          8 Info
```

0 **ALERT level A** = Most likely a serious problem - resolve or explain
0 **ALERT level B** = A potentially serious problem, consider carefully
5 **ALERT level C** = Check. Ensure it is not caused by an omission or oversight
6 **ALERT level G** = General information/check it is not something unexpected

0 ALERT type 1 CIF construction/syntax error, inconsistent or missing data
5 ALERT type 2 Indicator that the structure model may be wrong or deficient
2 ALERT type 3 Indicator that the structure quality may be low
2 ALERT type 4 Improvement, methodology, query or suggestion
2 ALERT type 5 Informative message, check

Validation response form

Please find below a validation response form (VRF) that can be filled in and pasted into your CIF.

```
# start Validation Reply Form
_vrf_PLAT094_p21c
;
PROBLEM: Ratio of Maximum / Minimum Residual Density ....       3.22 Report
RESPONSE: ...
;
_vrf_PLAT220_p21c
;
PROBLEM: Non-Solvent  Resd 1  C   Ueq(max)/Ueq(min) Range        4.2 Ratio
RESPONSE: ...
;
_vrf_PLAT222_p21c
;
PROBLEM: Non-Solv.  Resd 1  H   Uiso(max)/Uiso(min) Range        4.4 Ratio
RESPONSE: ...
;
_vrf_PLAT911_p21c
;
PROBLEM: Missing FCF Refl Between Thmin & STh/L=    0.600         75 Report
RESPONSE: ...
;
_vrf_PLAT971_p21c
;
PROBLEM: Check Calcd Resid. Dens.  1.03A   From C24             1.55 eA-3
RESPONSE: ...
;
# end Validation Reply Form
```

It is advisable to attempt to resolve as many as possible of the alerts in all categories. Often the minor alerts point to easily fixed oversights, errors and omissions in your CIF or refinement strategy, so attention to these fine details can be worthwhile. In order to resolve some of the more serious problems it may be necessary to carry out additional measurements or structure refinements. However, the purpose of your study may justify the reported deviations and the more serious of these should normally be commented upon in the discussion or experimental section of a paper or in the "special_details" fields of the CIF. checkCIF was carefully designed to identify outliers and unusual parameters, but every test has its limitations and alerts that are not important in a particular case may appear. Conversely, the absence of alerts does not guarantee there are no aspects of the results needing attention. It is up to the individual to critically assess their own results and, if necessary, seek expert advice.

Publication of your CIF in IUCr journals

A basic structural check has been run on your CIF. These basic checks will be run on all CIFs submitted for publication in IUCr journals (*Acta Crystallographica*, *Journal of Applied Crystallography*, *Journal of Synchrotron Radiation*); however, if you intend to submit to *Acta Crystallographica Section C* or *E* or *IUCrData*, you should make sure that full publication checks are run on the final version of your CIF prior to submission.

Publication of your CIF in other journals

Please refer to the *Notes for Authors* of the relevant journal for any special instructions relating to CIF submission.

PLATON version of 17/03/2019; check.def file version of 04/03/2019

Datablock p21c - ellipsoid plot

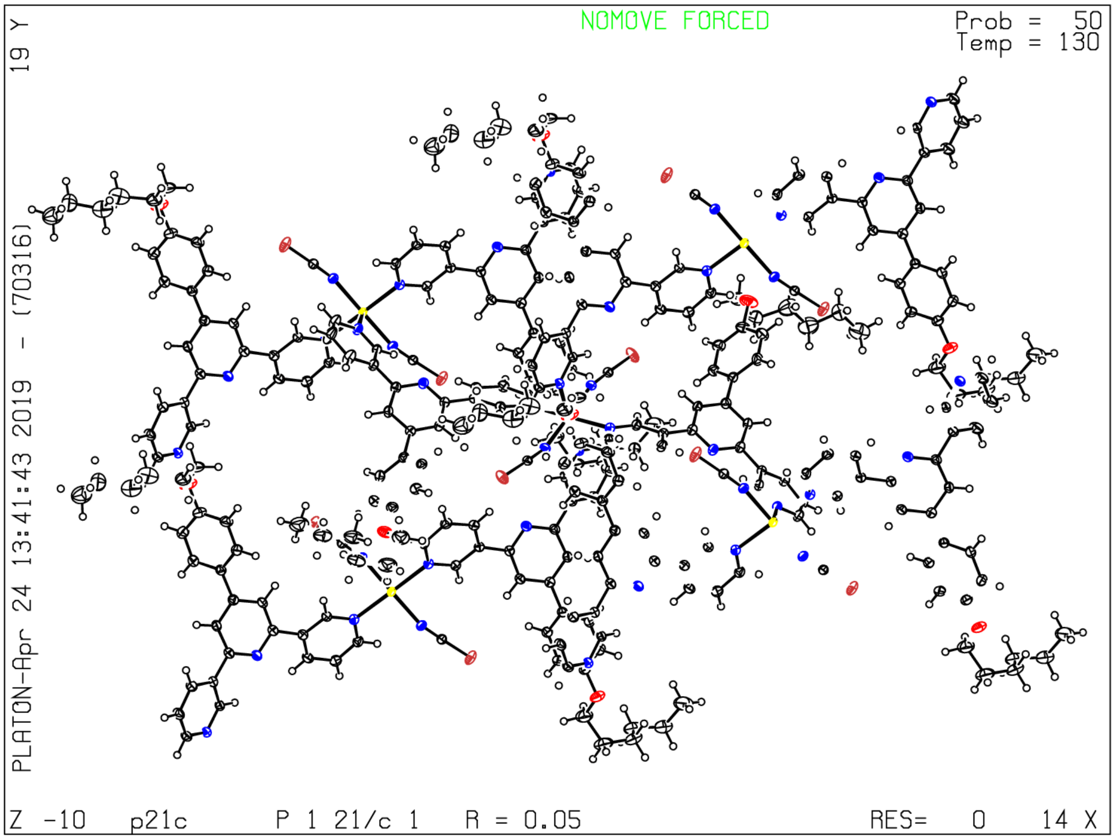

Supplement: Supplementary file 1 [file molecules-25-01663-s001.zip › ROD030A_checkcif.pdf]
