# Supplementary material for: Directing 2D-Coordination Networks: Combined Effects of a Conformationally Flexible 3,2′:6′,3″-Terpyridine and Chain Length Variation in 4′-(4-n-Alkyloxyphenyl) Substituents
Source: Molecules. 2020 Apr 4;25(7):1663. doi: 10.3390/molecules25071663 (PMC7180911; doi:10.3390/molecules25071663)

checkCIF/PLATON report

Structure factors have been supplied for datablock(s) rod011a_130k

THIS REPORT IS FOR GUIDANCE ONLY. IF USED AS PART OF A REVIEW PROCEDURE FOR PUBLICATION, IT SHOULD NOT REPLACE THE EXPERTISE OF AN EXPERIENCED CRYSTALLOGRAPHIC REFEREE.

No syntax errors found. CIF dictionary Interpreting this report

Datablock: rod011a_130k

Bond precision:	C-C = 0.0107 A		Wavelength=1.34143
Cell:	a=25.7185(3)	b=25.7185(3)	c=19.1253(3)
	alpha=90	beta=90	gamma=90
Temperature:	130 K		

	Calculated	Reported
Volume	12650.3(4)	12650.3(4)
Space group	P -4 21 c	P -4 21 c
Hall group	P -4 2n	P -4 2n
Moiety formula	C52 H46 Co N8 O2 S2, 4(C H Cl3)	C52 H46 Co N8 O2 S2, 4(C H Cl3)
Sum formula	C56 H50 Cl12 Co N8 O2 S2	C56 H52 Cl12 Co N8 O2 S2
Mr	1415.49	1417.50
Dx,g cm-3	1.486	1.489
Z	8	8
Mu (mm-1)	5.258	5.173
F000	5768.0	5784.0
F000’	5807.05	
h,k,lmax	32,32,23	30,32,23
Nref	12994[6999]	12678
Tmin,Tmax	0.354,0.696	0.954,0.975
Tmin’	0.227	

Correction method= # Reported T Limits: Tmin=0.954 Tmax=0.975
AbsCorr = MULTI-SCAN

Data completeness= 1.81/0.98 Theta(max)= 57.120

R(reflections)= 0.0626(9999) wR2(reflections)= 0.1710(12678)

S = 1.030 Npar= 733

The following ALERTS were generated. Each ALERT has the format
test-name_ALERT_alert-type_alert-level.
Click on the hyperlinks for more details of the test.

Alert level B

```
PLAT109_ALERT_2_B Twinning Matrix is inverted Laue group operation          ? Check
```

Alert level C

```
PLAT041_ALERT_1_C Calc. and Reported SumFormula    Strings  Differ     Please Check
PLAT043_ALERT_1_C Calculated and Reported Mol. Weight Differ by ..       2.01 Check
PLAT051_ALERT_1_C Mu(calc) and Mu(CIF) Ratio Differs from 1.0 by .       1.64 %
PLAT068_ALERT_1_C Reported F000 Differs from Calcd (or Missing)...     Please Check
PLAT094_ALERT_2_C Ratio of Maximum / Minimum Residual Density ....       2.30 Report
PLAT244_ALERT_4_C Low    Solvent Ueq as Compared to Neighbors of        C53 Check
PLAT244_ALERT_4_C Low    Solvent Ueq as Compared to Neighbors of        C54 Check
PLAT341_ALERT_3_C Low Bond Precision on  C-C Bonds ...............    0.01067 Ang.
PLAT601_ALERT_2_C Structure Contains Solvent Accessible VOIDS of .         86 Ang**3
PLAT911_ALERT_3_C Missing FCF Refl Between Thmin & STh/L=    0.600          4 Report
PLAT918_ALERT_3_C Reflection(s) with I(obs) much Smaller I(calc) .          2 Check
```

Alert level G

```
FORMU01_ALERT_1_G  There is a discrepancy between the atom counts in the
            _chemical_formula_sum and _chemical_formula_moiety. This is
            usually due to the moiety formula being in the wrong format.
            Atom count from _chemical_formula_sum:   C56 H52 Cl12 Co1 N8 O2 S2
            Atom count from _chemical_formula_moiety:C56 H50 Cl12 Co1 N8 O2 S2
FORMU01_ALERT_2_G  There is a discrepancy between the atom counts in the
            _chemical_formula_sum and the formula from the _atom_site* data.
            Atom count from _chemical_formula_sum:C56 H52 Cl12 Co1 N8 O2 S2
            Atom count from the _atom_site data:  C56 H50 Cl12 Co1 N8 O2 S2
ABSMU01_ALERT_1_G  Calculation of _exptl_absorpt_correction_mu
                not performed for this radiation type.
CELLZ01_ALERT_1_G Difference between formula and atom_site contents detected.
CELLZ01_ALERT_1_G WARNING: H atoms missing from atom site list. Is this intentional?
           From the CIF: _cell_formula_units_Z    8
           From the CIF: _chemical_formula_sum  C56 H52 Cl12 Co N8 O2 S2
           TEST: Compare cell contents of formula and atom_site data

           atom    Z*formula  cif sites diff
           C        448.00    448.00    0.00
           H        416.00    400.00   16.00
           Cl        96.00     96.00    0.00
           Co         8.00      8.00    0.00
           N         64.00     64.00    0.00
           O         16.00     16.00    0.00
           S         16.00     16.00    0.00
PLAT004_ALERT_5_G Polymeric Structure Found with Maximum Dimension          2 Info
PLAT072_ALERT_2_G SHELXL First  Parameter in WGHT  Unusually Large       0.12 Report
PLAT720_ALERT_4_G Number of Unusual/Non-Standard Labels ..........          1 Note
PLAT794_ALERT_5_G Tentative Bond Valency for Co1       (II)      .       1.87 Info
PLAT870_ALERT_4_G ALERTS Related to Twinning Effects Suppressed ..          ! Info
PLAT910_ALERT_3_G Missing # of FCF Reflection(s) Below Theta(Min).          1 Note
PLAT912_ALERT_4_G Missing # of FCF Reflections Above STh/L=  0.600         58 Note
PLAT916_ALERT_2_G Hooft y and Flack x Parameter Values Differ by .       0.46 Check
PLAT931_ALERT_5_G CIFcalcFCF  Twin Law ( 1 1 0)         Est.d BASF       0.46 Check
PLAT933_ALERT_2_G Number of OMIT Records in Embedded .res File ...          8 Note
PLAT950_ALERT_5_G Calculated (ThMax) and CIF-Reported Hmax Differ           2 Units
PLAT984_ALERT_1_G The  C-f’=    0.0148 Deviates from the B&C-Value     0.0137 Check
```

```
PLAT984_ALERT_1_G The Cl-f'=    0.3294 Deviates from the B&C-Value     0.3281 Check
PLAT984_ALERT_1_G The Co-f'=   -0.6673 Deviates from the B&C-Value    -0.6628 Check
PLAT984_ALERT_1_G The  N-f'=    0.0253 Deviates from the B&C-Value     0.0241 Check
PLAT984_ALERT_1_G The  O-f'=    0.0412 Deviates from the B&C-Value     0.0389 Check
PLAT985_ALERT_1_G The Cl-f"=    0.5404 Deviates from the B&C-Value     0.5435 Check
PLAT985_ALERT_1_G The Co-f"=    2.8829 Deviates from the B&C-Value     2.9049 Check
PLAT985_ALERT_1_G The  S-f"=    0.4248 Deviates from the B&C-Value     0.4295 Check
```

```
   0 ALERT level A = Most likely a serious problem - resolve or explain
   1 ALERT level B = A potentially serious problem, consider carefully
  11 ALERT level C = Check. Ensure it is not caused by an omission or oversight
  24 ALERT level G = General information/check it is not something unexpected

  16 ALERT type 1 CIF construction/syntax error, inconsistent or missing data
   7 ALERT type 2 Indicator that the structure model may be wrong or deficient
   4 ALERT type 3 Indicator that the structure quality may be low
   5 ALERT type 4 Improvement, methodology, query or suggestion
   4 ALERT type 5 Informative message, check
```

Validation response form

Please find below a validation response form (VRF) that can be filled in and pasted into your CIF.

```
# start Validation Reply Form
_vrf_PLAT041_rod011a_130k
;
PROBLEM: Calc. and Reported SumFormula    Strings  Differ     Please Check
RESPONSE: ...
;
_vrf_PLAT043_rod011a_130k
;
PROBLEM: Calculated and Reported Mol. Weight Differ by ..       2.01 Check
RESPONSE: ...
;
_vrf_PLAT051_rod011a_130k
;
PROBLEM: Mu(calc) and Mu(CIF) Ratio Differs from 1.0 by .       1.64 %
RESPONSE: ...
;
_vrf_PLAT068_rod011a_130k
;
PROBLEM: Reported F000 Differs from Calcd (or Missing)...     Please Check
RESPONSE: ...
;
_vrf_PLAT094_rod011a_130k
;
PROBLEM: Ratio of Maximum / Minimum Residual Density ....       2.30 Report
RESPONSE: ...
;
_vrf_PLAT244_rod011a_130k
;
PROBLEM: Low    Solvent Ueq as Compared to Neighbors of        C53 Check
RESPONSE: ...
;
_vrf_PLAT341_rod011a_130k
;
PROBLEM: Low Bond Precision on  C-C Bonds ...............    0.01067 Ang.
RESPONSE: ...
;
```

```
_vrf_PLAT601_rod011a_130k
;
PROBLEM: Structure Contains Solvent Accessible VOIDS of .         86 Ang**3
RESPONSE: ...
;
_vrf_PLAT911_rod011a_130k
;
PROBLEM: Missing FCF Refl Between Thmin & STh/L=    0.600          4 Report
RESPONSE: ...
;
_vrf_PLAT918_rod011a_130k
;
PROBLEM: Reflection(s) with I(obs) much Smaller I(calc) .          2 Check
RESPONSE: ...
;
# end Validation Reply Form
```

It is advisable to attempt to resolve as many as possible of the alerts in all categories. Often the minor alerts point to easily fixed oversights, errors and omissions in your CIF or refinement strategy, so attention to these fine details can be worthwhile. In order to resolve some of the more serious problems it may be necessary to carry out additional measurements or structure refinements. However, the purpose of your study may justify the reported deviations and the more serious of these should normally be commented upon in the discussion or experimental section of a paper or in the "special_details" fields of the CIF. checkCIF was carefully designed to identify outliers and unusual parameters, but every test has its limitations and alerts that are not important in a particular case may appear. Conversely, the absence of alerts does not guarantee there are no aspects of the results needing attention. It is up to the individual to critically assess their own results and, if necessary, seek expert advice.

Publication of your CIF in IUCr journals

A basic structural check has been run on your CIF. These basic checks will be run on all CIFs submitted for publication in IUCr journals (*Acta Crystallographica*, *Journal of Applied Crystallography*, *Journal of Synchrotron Radiation*); however, if you intend to submit to *Acta Crystallographica Section C* or *E* or *IUCrData*, you should make sure that full publication checks are run on the final version of your CIF prior to submission.

Publication of your CIF in other journals

Please refer to the *Notes for Authors* of the relevant journal for any special instructions relating to CIF submission.

PLATON version of 22/12/2019; check.def file version of 13/12/2019

Datablock rod011a_130k - ellipsoid plot

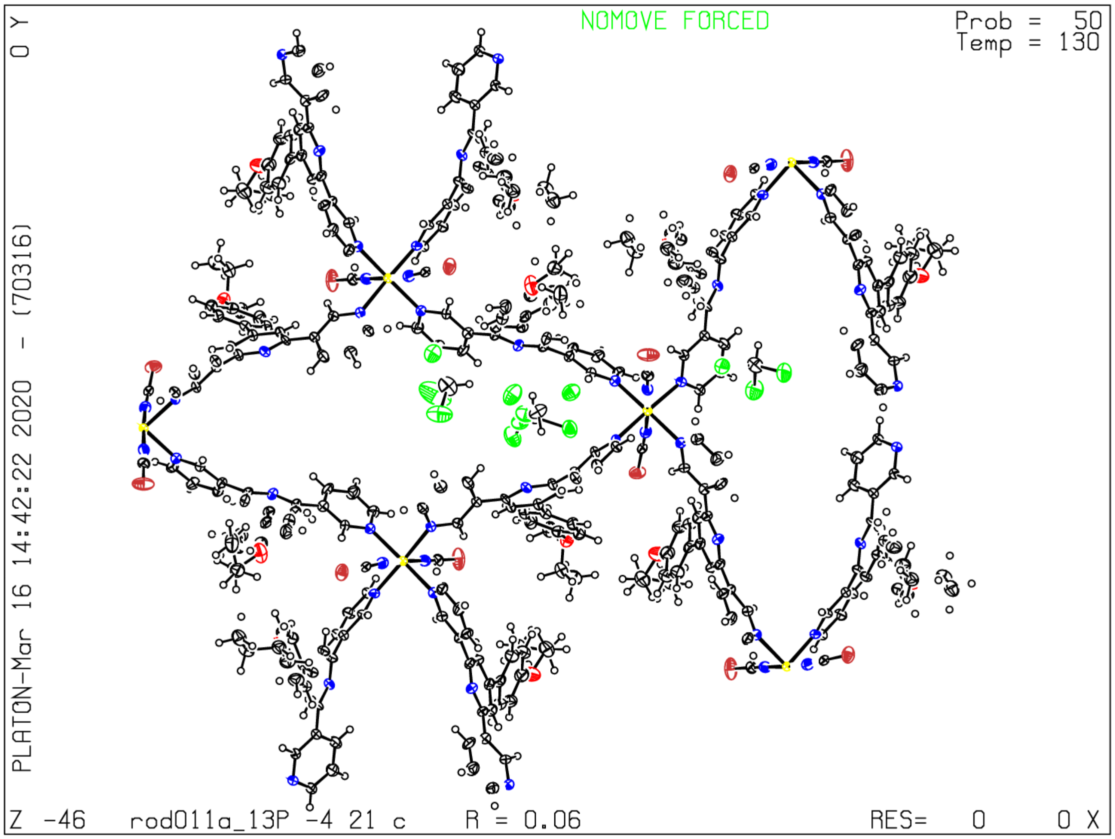

Supplement: Supplementary file 1 [file molecules-25-01663-s001.zip › ROD011A_130K_cifreport.pdf]
