# Supplementary material for: Directing 2D-Coordination Networks: Combined Effects of a Conformationally Flexible 3,2′:6′,3″-Terpyridine and Chain Length Variation in 4′-(4-n-Alkyloxyphenyl) Substituents
Source: Molecules. 2020 Apr 4;25(7):1663. doi: 10.3390/molecules25071663 (PMC7180911; doi:10.3390/molecules25071663)

checkCIF/PLATON report

Structure factors have been supplied for datablock(s) rod010aa_130k

THIS REPORT IS FOR GUIDANCE ONLY. IF USED AS PART OF A REVIEW PROCEDURE FOR PUBLICATION, IT SHOULD NOT REPLACE THE EXPERTISE OF AN EXPERIENCED CRYSTALLOGRAPHIC REFEREE.

No syntax errors found. CIF dictionary Interpreting this report

Datablock: rod010aa_130k

```
Bond precision:  C-C = 0.0110 A          Wavelength=1.34143

Cell:            a=25.3093(5)      b=25.3093(5)      c=18.1347(5)
                 alpha=90          beta=90           gamma=90
Temperature:     130 K
```

	Calculated	Reported
Volume	11616.4(6)	11616.4(6)
Space group	P 4/n c c	P 4/n c c
Hall group	-P 4a 2ac	-P 4a 2ac
Moiety formula	C48 H38 Co N8 O2 S2, 4(C0.20 H0.20 Cl0.60)	0.25(C192 H152 Co4 N32 O8 S8), 2.4(C H Cl3)
Sum formula	C50.40 H40.40 Cl7.20 Co N8 O2 S2	C50.40 H40.40 Cl7.20 Co N8 O2 S2
Mr	1168.40	1168.39
Dx,g cm-3	1.336	1.336
Z	8	8
Mu (mm-1)	4.327	4.267
F000	4769.6	4770.0
F000’	4795.26	
h,k,lmax	31,31,22	30,31,22
Nref	6061	6020
Tmin,Tmax	0.440,0.599	0.010,0.201
Tmin’	0.389	

```
Correction method= # Reported T Limits: Tmin=0.010 Tmax=0.201
AbsCorr = MULTI-SCAN

Data completeness= 0.993           Theta(max)= 57.467

R(reflections)= 0.0963( 3155)      wR2(reflections)= 0.3398( 6020)

S = 1.028                  Npar= 330
```

The following ALERTS were generated. Each ALERT has the format
test-name_ALERT_alert-type_alert-level.
Click on the hyperlinks for more details of the test.

Alert level B

```
PLAT230_ALERT_2_B Hirshfeld Test Diff for    C23      --C24      .       13.4 s.u.
```

Alert level C

```
RINTA01_ALERT_3_C  The value of Rint is greater than 0.12
            Rint given   0.146
PLAT020_ALERT_3_C The Value of Rint is Greater Than 0.12 .........      0.146 Report
PLAT051_ALERT_1_C Mu(calc) and Mu(CIF) Ratio Differs from 1.0 by .       1.41 %
PLAT084_ALERT_3_C High wR2 Value (i.e. > 0.25) ...................       0.34 Report
PLAT234_ALERT_4_C Large Hirshfeld Difference O1       --C20      .       0.19 Ang.
PLAT234_ALERT_4_C Large Hirshfeld Difference C19      --C20      .       0.21 Ang.
PLAT241_ALERT_2_C High   MainMol Ueq as Compared to Neighbors of         C4 Check
PLAT241_ALERT_2_C High   MainMol Ueq as Compared to Neighbors of        C23 Check
PLAT244_ALERT_4_C Low    Solvent Ueq as Compared to Neighbors of        C25 Check
PLAT260_ALERT_2_C Large Average Ueq of Residue Including       Cl1      0.152 Check
PLAT260_ALERT_2_C Large Average Ueq of Residue Including       Cl4      0.159 Check
PLAT341_ALERT_3_C Low Bond Precision on  C-C Bonds ...............    0.01105 Ang.
PLAT601_ALERT_2_C Structure Contains Solvent Accessible VOIDS of .         62 Ang**3
PLAT906_ALERT_3_C Large K Value in the Analysis of Variance ......      6.714 Check
PLAT906_ALERT_3_C Large K Value in the Analysis of Variance ......      2.133 Check
PLAT918_ALERT_3_C Reflection(s) with I(obs) much Smaller I(calc) .          2 Check
PLAT978_ALERT_2_C Number C-C Bonds with Positive Residual Density.          0 Info
```

Alert level G

```
ABSMU01_ALERT_1_G  Calculation of _exptl_absorpt_correction_mu
                not performed for this radiation type.
PLAT002_ALERT_2_G Number of Distance or Angle Restraints on AtSite          8 Note
PLAT003_ALERT_2_G Number of Uiso or Uij Restrained non-H Atoms ...          4 Report
PLAT004_ALERT_5_G Polymeric Structure Found with Maximum Dimension          2 Info
PLAT042_ALERT_1_G Calc. and Reported MoietyFormula Strings  Differ     Please Check
PLAT068_ALERT_1_G Reported F000 Differs from Calcd (or Missing)...     Please Check
PLAT072_ALERT_2_G SHELXL First  Parameter in WGHT  Unusually Large       0.17 Report
PLAT083_ALERT_2_G SHELXL Second Parameter in WGHT  Unusually Large      22.89 Why ?
PLAT171_ALERT_4_G The CIF-Embedded .res File Contains EADP Records          1 Report
PLAT176_ALERT_4_G The CIF-Embedded .res File Contains SADI Records          1 Report
PLAT186_ALERT_4_G The CIF-Embedded .res File Contains ISOR Records          1 Report
PLAT300_ALERT_4_G Atom Site Occupancy of Cl4        Constrained at        0.2 Check
PLAT300_ALERT_4_G Atom Site Occupancy of Cl5        Constrained at        0.2 Check
PLAT300_ALERT_4_G Atom Site Occupancy of Cl6        Constrained at        0.2 Check
PLAT300_ALERT_4_G Atom Site Occupancy of C26        Constrained at        0.2 Check
PLAT300_ALERT_4_G Atom Site Occupancy of H26        Constrained at        0.2 Check
PLAT302_ALERT_4_G Anion/Solvent/Minor-Residue Disorder (Resd  3  )       100% Note
PLAT432_ALERT_2_G Short Inter X...Y Contact  Cl6      ..C26              3.24 Ang.
                                         -1/2+y,1-x,1-z  =     11_566 Check
PLAT434_ALERT_2_G Short Inter HL..HL Contact Cl1      ..Cl1              3.03 Ang.
                                     -1/2+y,1/2+x,3/2-z  =      7_456 Check
PLAT794_ALERT_5_G Tentative Bond Valency for Co1       (II)      .       1.96 Info
PLAT860_ALERT_3_G Number of Least-Squares Restraints .............         39 Note
PLAT910_ALERT_3_G Missing # of FCF Reflection(s) Below Theta(Min).          1 Note
PLAT912_ALERT_4_G Missing # of FCF Reflections Above STh/L=  0.600         40 Note
PLAT933_ALERT_2_G Number of OMIT Records in Embedded .res File ...          5 Note
PLAT984_ALERT_1_G The  C-f'=    0.0148 Deviates from the B&C-Value     0.0137 Check
PLAT984_ALERT_1_G The Cl-f'=    0.3294 Deviates from the B&C-Value     0.3281 Check
PLAT984_ALERT_1_G The Co-f'=   -0.6673 Deviates from the B&C-Value    -0.6628 Check
```

```
PLAT984_ALERT_1_G The  N-f'=    0.0253 Deviates from the B&C-Value     0.0241 Check
PLAT984_ALERT_1_G The  O-f'=    0.0412 Deviates from the B&C-Value     0.0389 Check
PLAT985_ALERT_1_G The Cl-f"=    0.5404 Deviates from the B&C-Value     0.5435 Check
PLAT985_ALERT_1_G The Co-f"=    2.8829 Deviates from the B&C-Value     2.9049 Check
```

```
   0 ALERT level A = Most likely a serious problem - resolve or explain
   1 ALERT level B = A potentially serious problem, consider carefully
  17 ALERT level C = Check. Ensure it is not caused by an omission or oversight
  31 ALERT level G = General information/check it is not something unexpected

  11 ALERT type 1 CIF construction/syntax error, inconsistent or missing data
  14 ALERT type 2 Indicator that the structure model may be wrong or deficient
   9 ALERT type 3 Indicator that the structure quality may be low
  13 ALERT type 4 Improvement, methodology, query or suggestion
   2 ALERT type 5 Informative message, check
```

Validation response form

Please find below a validation response form (VRF) that can be filled in and pasted into your CIF.

```
# start Validation Reply Form
_vrf_RINTA01_rod010aa_130k
;
PROBLEM: The value of Rint is greater than 0.12
RESPONSE: ...
;
_vrf_PLAT020_rod010aa_130k
;
PROBLEM: The Value of Rint is Greater Than 0.12 .........      0.146 Report
RESPONSE: ...
;
_vrf_PLAT051_rod010aa_130k
;
PROBLEM: Mu(calc) and Mu(CIF) Ratio Differs from 1.0 by .       1.41 %
RESPONSE: ...
;
_vrf_PLAT084_rod010aa_130k
;
PROBLEM: High wR2 Value (i.e. > 0.25) ...................       0.34 Report
RESPONSE: ...
;
_vrf_PLAT234_rod010aa_130k
;
PROBLEM: Large Hirshfeld Difference O1       --C20      .       0.19 Ang.
RESPONSE: ...
;
_vrf_PLAT241_rod010aa_130k
;
PROBLEM: High   MainMol Ueq as Compared to Neighbors of         C4 Check
RESPONSE: ...
;
_vrf_PLAT244_rod010aa_130k
;
PROBLEM: Low    Solvent Ueq as Compared to Neighbors of        C25 Check
RESPONSE: ...
;
_vrf_PLAT260_rod010aa_130k
;
PROBLEM: Large Average Ueq of Residue Including       Cl1      0.152 Check
```

```
RESPONSE: ...
;
_vrf_PLAT341_rod010aa_130k
;
PROBLEM: Low Bond Precision on  C-C Bonds ..............    0.01105 Ang.
RESPONSE: ...
;
_vrf_PLAT601_rod010aa_130k
;
PROBLEM: Structure Contains Solvent Accessible VOIDS of .         62 Ang**3
RESPONSE: ...
;
_vrf_PLAT906_rod010aa_130k
;
PROBLEM: Large K Value in the Analysis of Variance ......      6.714 Check
RESPONSE: ...
;
_vrf_PLAT918_rod010aa_130k
;
PROBLEM: Reflection(s) with I(obs) much Smaller I(calc) .          2 Check
RESPONSE: ...
;
_vrf_PLAT978_rod010aa_130k
;
PROBLEM: Number C-C Bonds with Positive Residual Density.          0 Info
RESPONSE: ...
;
# end Validation Reply Form
```

It is advisable to attempt to resolve as many as possible of the alerts in all categories. Often the minor alerts point to easily fixed oversights, errors and omissions in your CIF or refinement strategy, so attention to these fine details can be worthwhile. In order to resolve some of the more serious problems it may be necessary to carry out additional measurements or structure refinements. However, the purpose of your study may justify the reported deviations and the more serious of these should normally be commented upon in the discussion or experimental section of a paper or in the "special_details" fields of the CIF. checkCIF was carefully designed to identify outliers and unusual parameters, but every test has its limitations and alerts that are not important in a particular case may appear. Conversely, the absence of alerts does not guarantee there are no aspects of the results needing attention. It is up to the individual to critically assess their own results and, if necessary, seek expert advice.

Publication of your CIF in IUCr journals

A basic structural check has been run on your CIF. These basic checks will be run on all CIFs submitted for publication in IUCr journals (*Acta Crystallographica*, *Journal of Applied Crystallography*, *Journal of Synchrotron Radiation*); however, if you intend to submit to *Acta Crystallographica Section C* or *E* or *IUCrData*, you should make sure that full publication checks are run on the final version of your CIF prior to submission.

Publication of your CIF in other journals

Please refer to the *Notes for Authors* of the relevant journal for any special instructions relating to CIF submission.

PLATON version of 22/12/2019; check.def file version of 13/12/2019

Datablock rod010aa_130k - ellipsoid plot

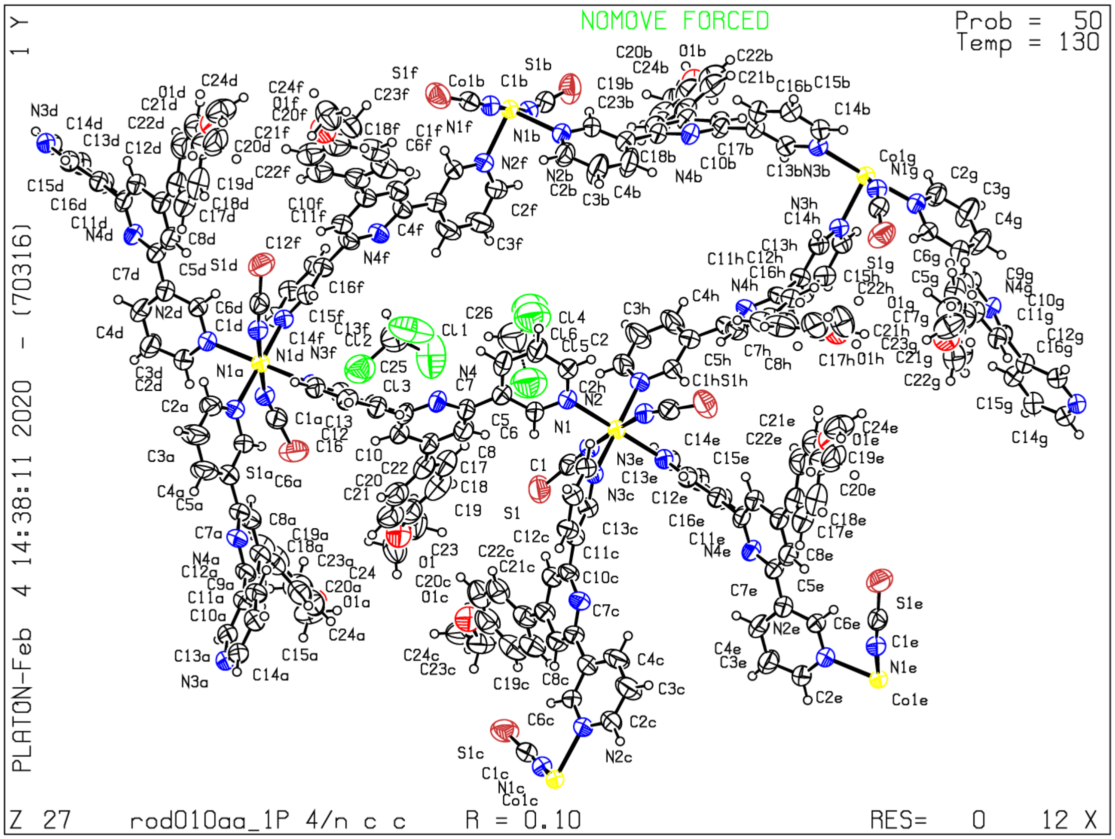

Supplement: Supplementary file 1 [file molecules-25-01663-s001.zip › ROD010AA_130K_checkcif.pdf]
